# *Candida albicans* colonization modulates murine ethanol consumption and behavioral responses through elevation of serum prostaglandin E_2_ and impact on the striatal dopamine system

**DOI:** 10.1128/mbio.02239-25

**Published:** 2025-10-16

**Authors:** Andrew W. Day, Emma Hayes, Jeyra Perez-Lozada, Alyssa DiLeo, Katrina Blandino, Jamie Maguire, Carol A. Kumamoto

**Affiliations:** 1 Graduate School of Biomedical Sciences, Tufts University, Boston, Massachusetts, USA; 2 Department of Molecular Biology and Microbiology, Tufts University, Boston, Massachusetts, USA; 3 Department of Neuroscience, Tufts University 146186, Boston, Massachusetts, USA; Biomedicine Discovery Institute, Clayton, Victoria, Australia

**Keywords:** *Candida albicans*, GI colonization, gut-brain axis, ethanol consumption, prostaglandin E2, dopamine receptors

## Abstract

*Candida albicans* is a commensal yeast that is a common component of the gastrointestinal (GI) microbiome of humans. *C. albicans* has been shown to bloom in the GI tract of individuals with alcohol use disorder (AUD) and can promote and increase the severity of alcoholic liver disease. However, the effects of *C. albicans* blooms on the host in the context of AUD or AUD-related phenotypes, such as ethanol preference, have been unstudied. In this work, we report a reduction in ethanol consumption and preference in mice colonized with *C. albicans. C. albicans-*colonized mice exhibited elevated levels of serum prostaglandin E_2_ (PGE_2_), and the reduced ethanol preference was reversed by injection with antagonists of PGE_2_ receptors. Furthermore, injection of mice with a PGE_2_ derivative decreased their ethanol preference. These results show that PGE_2_ acting on its receptors prostaglandin E receptor 1 (EP1) and prostaglandin E receptor 2 (EP2) drives reduced ethanol preference in *C. albicans-*colonized mice. We also showed altered transcription of dopamine receptors in the dorsal striatum of *C. albicans-*colonized mice and more rapid acquisition of ethanol-conditioned taste aversion, suggesting alterations to reinforcement or aversion learning. Finally, *C. albicans*-colonized mice were more susceptible to ethanol-induced motor coordination impairment, showing significant alterations to the behavioral effects of ethanol. This study identifies a member of the fungal microbiome that alters ethanol preference and demonstrates a role for PGE_2_ signaling in these phenotypes.

**IMPORTANCE:**

*Candida albicans* is a commensal yeast that is found in the gut of most individuals. *C. albicans* has been shown to contribute to alcoholic liver disease. Outside of this, the impact of intestinal fungi on alcohol use disorder (AUD) had been unstudied. As AUD is a complex disorder characterized by high relapse rates and there are only three FDA-approved therapies for the maintenance of abstinence, it is important to study novel AUD contributors to find new therapeutic targets. Here, we show that an intestinal fungus, *C. albicans*, can alter mammalian ethanol consumption through an immune modulator, prostaglandin E_2_. The results highlight novel contributors to AUD-related phenotypes and further implicate the gut-brain axis in AUD. Future studies could lead to new therapeutic avenues for the treatment of AUD.

## INTRODUCTION

*Candida albicans* is a commensal fungus that is found in the gastrointestinal (GI) tract of roughly 65% of individuals (1), although colonization abundance shows considerable variation throughout different populations and data sets. *C. albicans* is an opportunistic pathogen that can cause oral thrush, vulvovaginal candidiasis, and life-threatening bloodstream infections (2–4). Because of the severity of these infections, *C. albicans* pathogenesis has been extensively investigated. However, prior to infections, *C. albicans* lives in the gut and can breach the GI tract to infect the host (5–7).

One host setting associated with enrichment for *C. albicans* in the GI tract is alcohol use disorder (AUD), a condition in which individuals have lost the ability to control or stop their alcohol consumption (8). Estimates suggest roughly 2.3 billion people in the world consume alcohol, and over 5% of the world’s adult population has an AUD (9). *C. albicans* is enriched in the fecal microbiome in individuals with AUD (10–12) and contributes to the progression and severity of alcoholic liver disease (ALD) (10, 11). Immune responses to intestinal fungi correlate with a reduced 5 year survival in those with ALD (10), showing that changes in abundance of intestinal fungi have vital consequences to the host.

The gut microbiota can affect host behavior via effects on the gut-brain axis (GBA). The GBA is defined as the bidirectional communication between the GI tract and the brain (13, 14). The GBA has been implicated in many disorders ranging from autism to addiction (15–18). Initial work characterizing the GBA in AUD found dysbiosis in the GI tract can contribute to depression and craving (19). Specific bacteria have been found that contribute to alcohol craving (20, 21). Furthermore, fecal microbiota transplants in mice and humans can modulate ethanol preference and craving, respectively (22, 23). These findings support the model that changes to the microbiome in AUD can regulate ethanol consumption.

As a mechanism linking microbiome changes to the GBA in AUD, we investigated the role of prostaglandin E_2_ (PGE_2_), an eicosanoid compound synthesized from arachidonic acid. A previous study showed that treatment with PGE_2_ reduced ethanol consumption in rats (24). Serum PGE_2_ levels have previously been shown to be higher in antibiotic-treated mice with GI colonization by *C. albicans* (25). Furthermore, we previously showed that *C. albicans* colonization in mice dysregulated endocannabinoids (26), another set of compounds that utilize arachidonic acid as a precursor (27). The arachidonic acid-derived host lipidome is thus altered in *C. albicans-*colonized mice. We therefore tested for a role for PGE_2_ in reducing ethanol consumption in *C. albicans-*colonized mice.

In this study, we show that *C. albicans* GI colonization in mice reduced their ethanol consumption. *C. albicans*-colonized mice had elevated serum PGE_2_ compared to mock-colonized mice, and injection of uncolonized mice with dimethyl-PGE_2_ reduced ethanol consumption. The reduction in ethanol consumption in *C. albicans-*colonized mice was reversed by antagonizing receptors for PGE_2_. *C. albicans*-colonized mice exhibited altered expression of dopamine receptors in the dorsal striatum, a more rapid acquisition of ethanol-induced conditioned taste aversion (CTA), and were more sensitive to ethanol-induced motor coordination deficits, which could be reversed by PGE_2_ receptor antagonist treatment. These results show for the first time that changes to the fungal microbiome can alter ethanol preference and ethanol-induced behavior in mice.

## RESULTS

### *C. albicans* colonization in female mice reduces ethanol consumption

**Fig 1:**
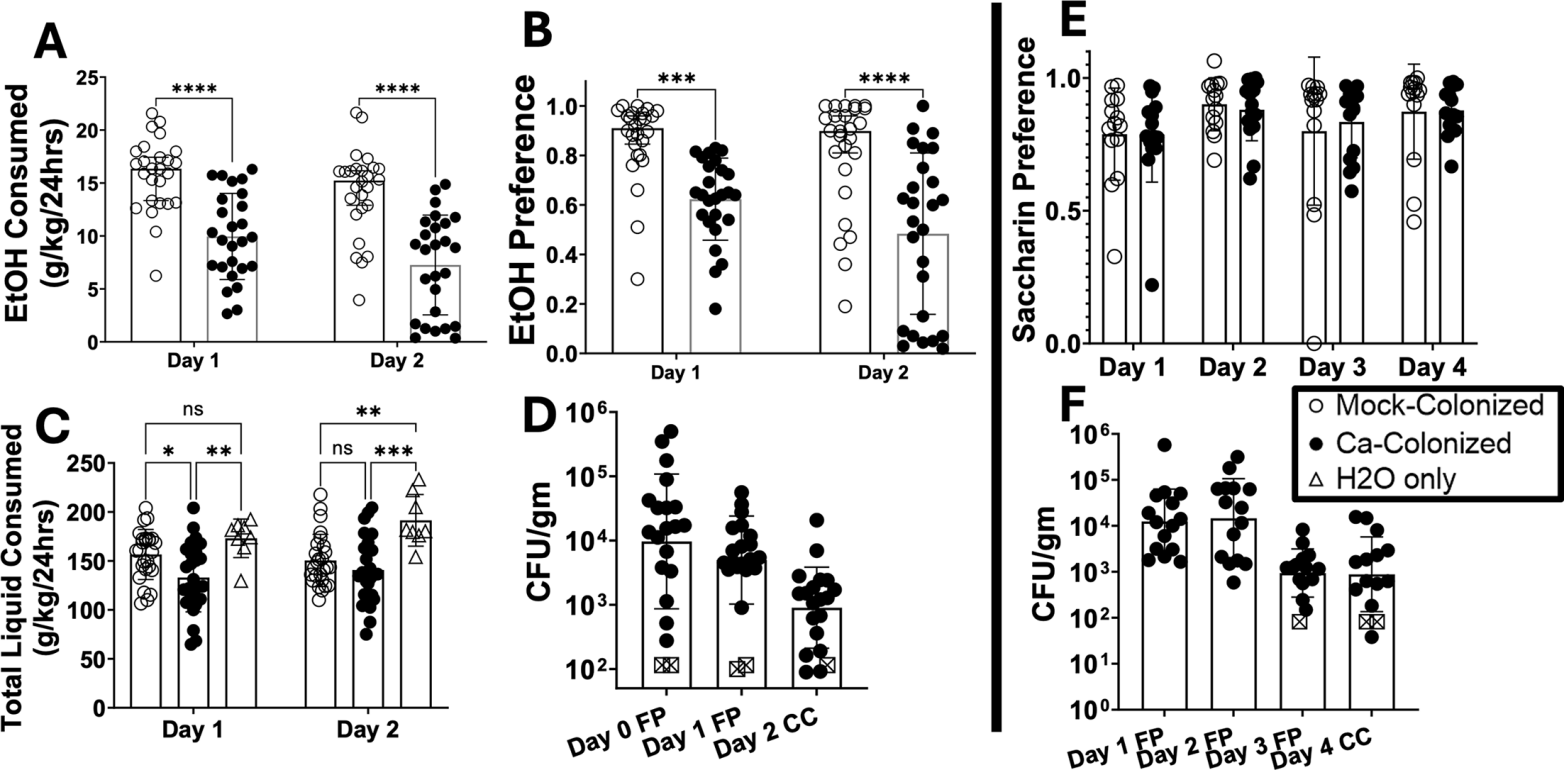
*C. albicans*-colonized mice consume significantly less ethanol and have lower ethanol preference than mock-colonized mice. Single-housed female C57BL/6 mice were orally inoculated with *C. albicans* strain CKY101 or phosphate-buffered saline. After inoculation, mice were subjected to a two-bottle choice experiment, with one bottle of 15% (vol/vol) ethanol and one bottle of water. Liquid consumption was measured by weighing the bottles daily. (**A**) Ethanol consumption (grams of ethanol per kilogram of body weight) on the 1st and 2nd day of access to ethanol. Mock-colonized (open circles), *C. albicans-*colonized (black circles). (**B**) Ethanol preference (volume of ethanol solution consumed per total volume of liquid consumed). Mock-colonized (open circles), *C. albicans*-colonized (black circles). (**C**) Total liquid consumed per day (grams of total liquid per kilogram of body weight). Mock or *C. albicans*-colonized mice (open and black circles, respectively) and mice that were only given access to water (H_2_O-only, triangles). (**D**) Colony forming units (CFU) per gram of fecal pellet on each day of collection. Black circles show individual mice, and open squares show values below the limit of detection. Geometric means are shown by bars, and geometric standard deviation is shown by error bars. (**E**) Saccharin preference (volume of saccharin solution per total volume of liquid consumed). (**F**) CFU per gram of fecal pellets from the saccharin preference experiment. Symbols as in panel D. (**A–C, E**) Boxes represent mean values, error bars show standard deviation, and each symbol represents an individual mouse. A two-way analysis of variance corrected for repeated measures was performed for statistics (*P = 0.0144; **P < 0.0035; ***P < 0.0003; ****P < 0.0001).

Mice were orally inoculated with *C. albicans* as described (26). We previously showed that inoculation did not result in significant changes in bacterial microbiota composition and did not evoke significant inflammation (26). Colonized and mock-colonized mice were subjected to a continuous-access two-bottle choice experiment, where mice were given access to two sipper tubes containing water or 15% ethanol simultaneously as described in Materials and Methods. Ethanol consumption and ethanol preference showed reductions on both days of the protocol in *C. albicans*-colonized mice compared to mock-colonized mice (mock) (Fig. 1A and B). *C. albicans*-colonized mice consumed slightly less total liquid than mock on day 1 and the same amount of liquid on day 2. Both groups drank less total liquid than mice only given access to water (H_2_O-only) (Fig. 1C). Colony-forming units per gram of fecal pellets (FP) or cecum contents (CC) demonstrated the presence of *C. albicans* in the GI tract of inoculated mice throughout the experiment (Fig. 1D). From these results, we concluded that *C. albicans* colonization reduced ethanol consumption in female mice.

Saccharin preference was tested to determine whether *C. albicans*-colonized mice showed alterations in preference to other rewarding substances (28). Saccharin preference was measured in a similar continuous-access two-bottle choice experiment as described in Materials and Methods. Saccharin preference per day showed no effect of *C. albicans* colonization (Fig. 1E). Colony forming units (CFU per gram of FP or CC showed colonization throughout the experiment (Fig. 1F). There were no differences to total liquid consumption between mock-colonized and *C. albicans*-colonized mice in these experiments (Fig. S1). These results showed that *C. albicans* colonization did not exert broad effects on consumption of all rewarding substances. Rather, the effects were more selective.

### *C. albicans***-**colonized mice have elevated serum PGE_2_, and PGE_2_ injection reduces murine ethanol preference

**Fig 2:**
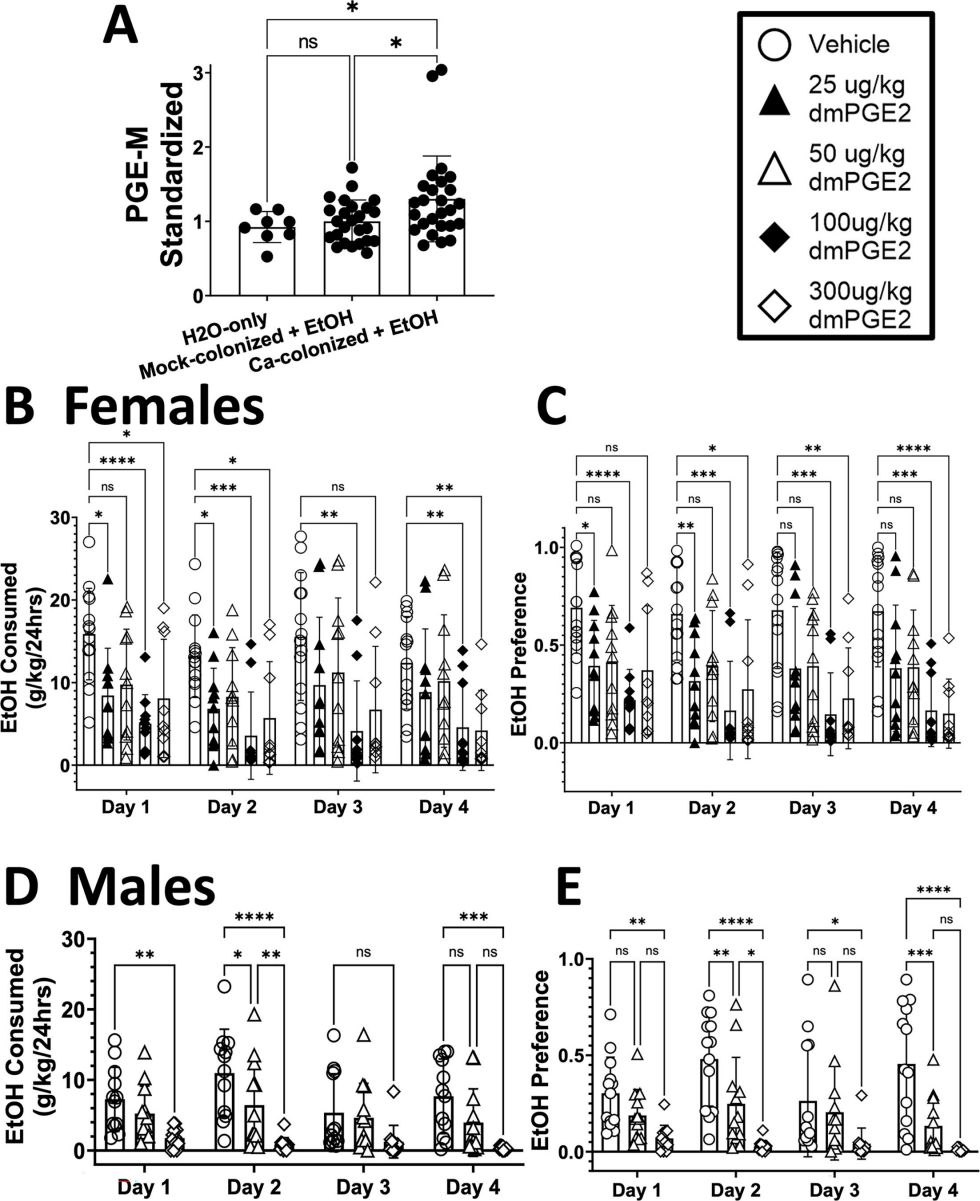
*C. albicans*-colonized mice have elevated serum PGE_2_, and injection of 16,16-dimethyl PGE_2_ (dmPGE_2_) reduced ethanol consumption. Single-housed female C57BL/6 mice were orally inoculated with *C. albicans* strain CKY101 or phosphate-buffered saline and subjected to the two-bottle choice experiment as in Fig. 1. On day 2, mice were euthanized, and serum was collected. (**A**) PGE-metabolite concentration, standardized to the average in mock-colonized mice within each experiment. Mice only given access to H_2_O (left bar), mock-colonized mice that consumed ethanol (middle bar), *C. albicans*-colonized mice that consumed ethanol (right bar). Bars show mean, error bars show standard deviation, and each symbol indicates an individual mouse. One-way analysis of variance (ANOVA) was performed for statistical analysis (*, *P* < 0.0354). (**B, D**) Single-housed female (**B**) or male (**D**) C57BL/6 mice were injected subcutaneously once per day with dmPGE_2_ (concentrations as shown) or vehicle and subjected to the two-bottle choice experiment as in Fig. 1. On day 4, mice were euthanized, and brains were collected. Ethanol consumed per day (grams of ethanol per kilogram of body weight) is shown. (**C, E**) Daily ethanol preference (volume ethanol solution consumed per total volume liquid consumed) is shown. C shows females, and E shows males. (**B–E**) Mean and standard deviation are shown. Each symbol represents one mouse. A two-way ANOVA test corrected for repeated measures was used for statistical significance. ns, *P* < 0.0939; *P < 0.0499; **P < 0.0100; ***P < 0.0011; ****, *P* < 0.0001.

Levels of PGE_2_ were measured in serum obtained from *C. albicans*-colonized or mock-colonized mice following 2 days of ethanol consumption. Since PGE_2_ is not a stable molecule, concentrations of a metabolite of PGE_2_, 13,14-dihydro-15-keto PGE_2_ (PGE-m), commonly utilized as a marker for PGE_2_ (29), were assayed by enzyme-linked immunosorbent assay (ELISA). The assay showed that *C. albicans*-colonized mice had elevated serum PGE-m levels compared to H_2_O-only or mock groups (Fig. 2A).

To test the effect of elevated PGE_2_ on ethanol consumption, a 4 day, two-bottle choice experiment was performed in which mice were injected with 16,16-dimethyl PGE_2_ (dmPGE_2_), a commonly used, stable derivative of PGE_2_ (30). Mice received either vehicle or one of four different concentrations of dmPGE_2_ (25, 50, 100, 300 µg/kg) by subcutaneous injection each day. In female mice, lower concentrations (25 or 50 µg/kg) of dmPGE_2_ led to low-level decreases in ethanol preference and consumption, and higher concentrations led to larger decreases compared to phosphate-buffered saline (PBS)-injected mice (Fig. 2B and C). In males, we observed a similar effect (Fig. 2D and E) with 50 µg/kg of dmPGE_2_ leading to small changes and 300 µg/kg of dmPGE_2_ having a significant effect. Additionally, we noted that ethanol consumption in vehicle-injected mice was lower in males than in females. Thus, dmPGE_2_ injection in mice decreased ethanol consumption in a dose-dependent manner, supporting the model that elevation of endogenous PGE_2_ results in decreased ethanol consumption.

### EP receptor antagonism increases ethanol consumption and preference to mock-colonized levels

**Fig 3:**
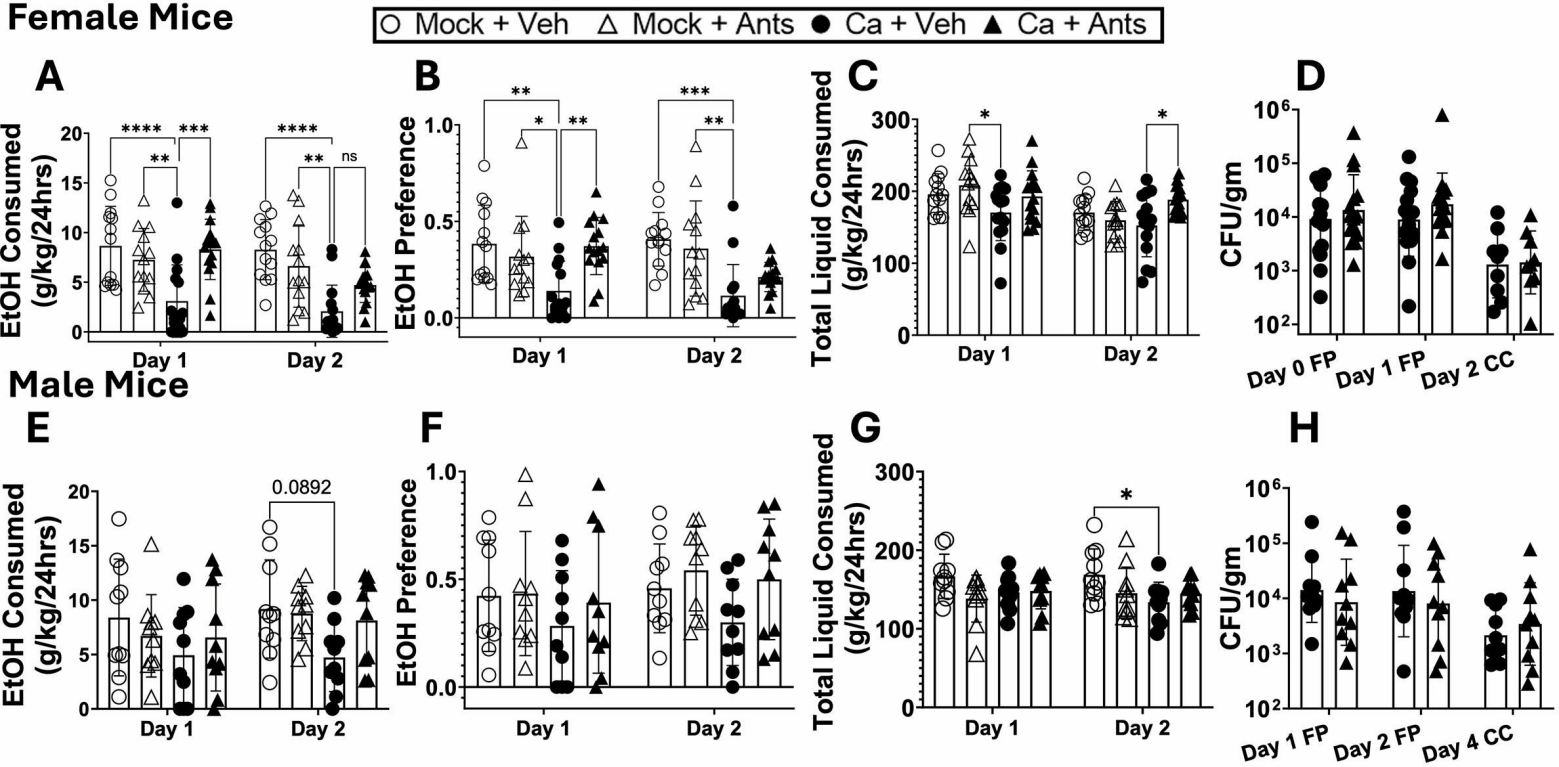
EP receptor antagonism elevates *C. albicans*-colonized mouse ethanol consumption to mock-colonized levels in female and male mice. Single-housed female C57BL/6 mice (**A–D**) or male C57BL/6 mice (**E–H**) were orally inoculated with *C. albicans* strain CKY101 or PBS, injected subcutaneously with EP1 antagonist (SC-51089, 2 g/kg) and EP2 antagonist (TG6-10-1, 2 g/kg) or vehicle, and subjected to the two-bottle choice experiment as in Fig. 1. Females were euthanized on day 2, males on day 4. (**A, E**) Ethanol consumption, (**B, F**) ethanol preference, (**C, G**) total liquid consumed per day. (**A–C, E–G**) Mock-colonized mice with vehicle (open circles) or antagonists (open triangles), *C. albicans*-colonized mice with vehicle (black circles) or antagonists (black triangles). Boxes represent mean values, error bars show standard deviation, and each symbol represents an individual mouse. A two-way analysis of variance (ANOVA) corrected for repeated measures was performed for statistics. ns, *P* = 0.1182; *P < 0.0368; **P < 0.0054; ***P = 0.0001; ****P < 0.0001. (**D, H**) Shows CFU per gram of fecal pellet on each day of collection. Black circles (*C. albicans* with vehicle) or black triangles (*C. albicans* with antagonists) show individual mice. Geometric means are shown by bars, and geometric standard deviation is shown by error bars. A two-way ANOVA was performed for statistics.

Based on these results, we hypothesized that PGE_2_ is a key driver of reduced ethanol preference in *C. albicans*-colonized mice. To test this hypothesis, female mice were orally inoculated with *C. albicans* or mock-colonized with PBS and injected with 2 g/kg each of an antagonist of prostaglandin E receptor 1 (EP1) (SC-51089, Cayman Chemical) and an antagonist of prostaglandin E receptor 2 (EP2) antagonist (TG6-10-1, Cayman Chemical) subcutaneously each day of the 2 day two-bottle choice experiment. Mock-colonized mice injected with vehicle or antagonist showed no difference in ethanol consumption or preference (Fig. 3A and B). Mice that were colonized with *C. albicans* and injected with vehicle showed significantly lower ethanol consumption and preference compared to other groups. Those injected with antagonists showed significantly higher ethanol consumption and preference on day 1 and a trend toward an increase in ethanol consumption on day 2. Some reductions in total liquid consumed in *C. albicans*-colonized, vehicle-injected mice were observed (Fig. 3C). Mice had stable colonization throughout the experiment (Fig. 3D).

We also tested whether male mice had a reduction in ethanol consumption following *C. albicans* colonization and whether this phenotype could be reversed by EP antagonists. A trend toward reduction in ethanol consumption by the vehicle-injected, *C. albicans*-colonized mice was observed on day 2 of ethanol consumption, and total liquid consumed was also reduced (Fig. 3E, F, and G). These mice had stable colonization throughout the experiment (Fig. 3H). These results support the model that activation of EP1/EP2 receptors in *C. albicans*-colonized mice decreases their ethanol consumption and preference. Further experiments were conducted with females only since females showed strong effects of colonization.

### Increased serum PGE_2_ in *C. albicans***-**colonized mice correlates with gene expression in the brain

We next tested the hypothesis that *C. albicans* gut colonization and elevation of PGE_2_ concentration would result in transcriptomic changes in the brain. PGE_2_ binds to its cognate receptors encoded by four different genes, *Ep1*, *Ep2*, *Ep3*, and *Ep4* (31). PGE_2_ has been shown to upregulate expression of *Ep* receptor genes in a dose-dependent manner in neuronal cell lines and various tissues in the body (32, 33). We decided to focus on a region of the brain involved in habit formation, the dorsal striatum (DS) (34), as well as other addiction-implicated regions such as the prefrontal cortex (PFC) and nucleus accumbens (NAc) (35).

**Fig 4:**
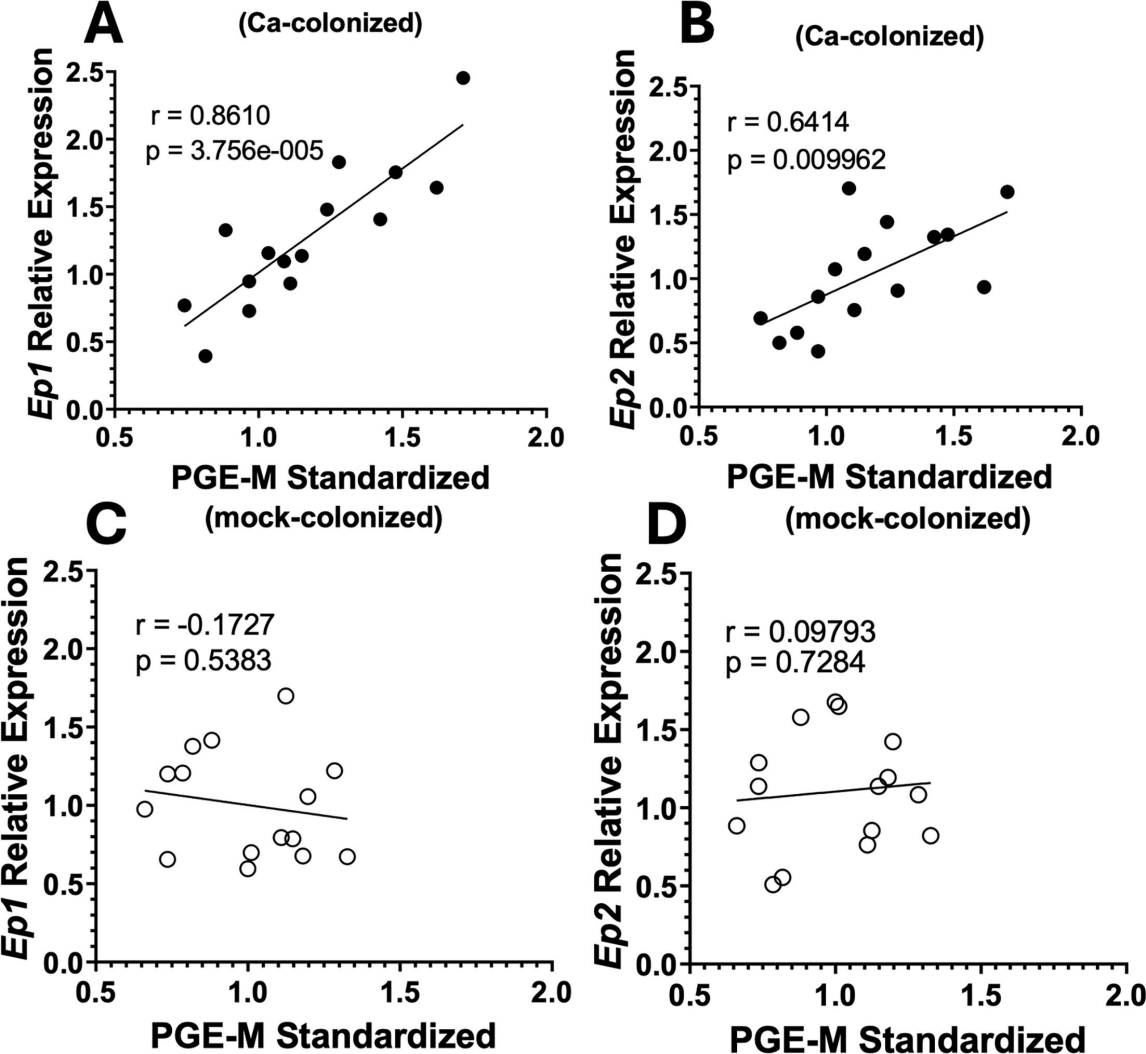
Serum PGE-metabolite correlates with EP receptor expression in the dorsal striatum in *C. albicans*-colonized mice. Single-housed female C57BL/6 mice were orally inoculated with *C. albicans* strain CKY101 or PBS and subjected to the two-bottle choice experiment as in Fig. 1. On day 2, mice were euthanized, and brain and serum were collected. Expression of *Ep* genes relative to average expression in mock-colonized mice from the same experimental trial, plotted as a function of concentration of PGE-metabolite standardized to the average in mock-colonized mice in the same experimental trial. Each symbol represents an individual mouse. (**A, B**) *C. albicans*-colonized mice (black circles), (**C, D**) mock-colonized mice (open circles). (**A, C**) *Ep1,* (**B, D**) *Ep2*. Pearson correlations were used to test for significant correlations, *r* = correlation strength and *P* = statistical significance. Significant correlations were observed between *Ep1* and *Ep2* expressions and concentration of PGE-m in *C. albicans-*colonized mice.

To test the hypothesis, reverse transcription-quantitative polymerase chain reaction (RT-qPCR) from DS-, PFC-, and NAc-cDNA was performed from *C. albicans-* and mock-colonized mice, and expression of *Ep1* and *Ep2* relative to mock-colonized from the same experimental trial was measured. There were no significant differences in *Ep* receptor expression between groups in the DS, PFC, or NAc (Fig. S2). However, correlations between PGE-m concentration in the serum and relative expression of *Ep1* or *Ep2* in the DS of the same *C. albicans*-colonized mouse were observed. Gene expression relative to the average for mock-colonized was plotted against the concentration of PGE-m (also standardized to mock-colonized). Figure 4A and B demonstrates that mice with higher concentrations of serum PGE_2_ exhibited higher expression of *Ep1* and *Ep2*. In mock-colonized mice, there were no significant correlations (Fig. 4C and D). We observed similar significant correlations of *Ep1* expression and PGE-m concentration in the PFC and NAc (Fig. S3). Elevated PGE_2_ levels were thus associated with higher expression of *Ep* receptors in multiple regions of the brain.

**Fig 5:**
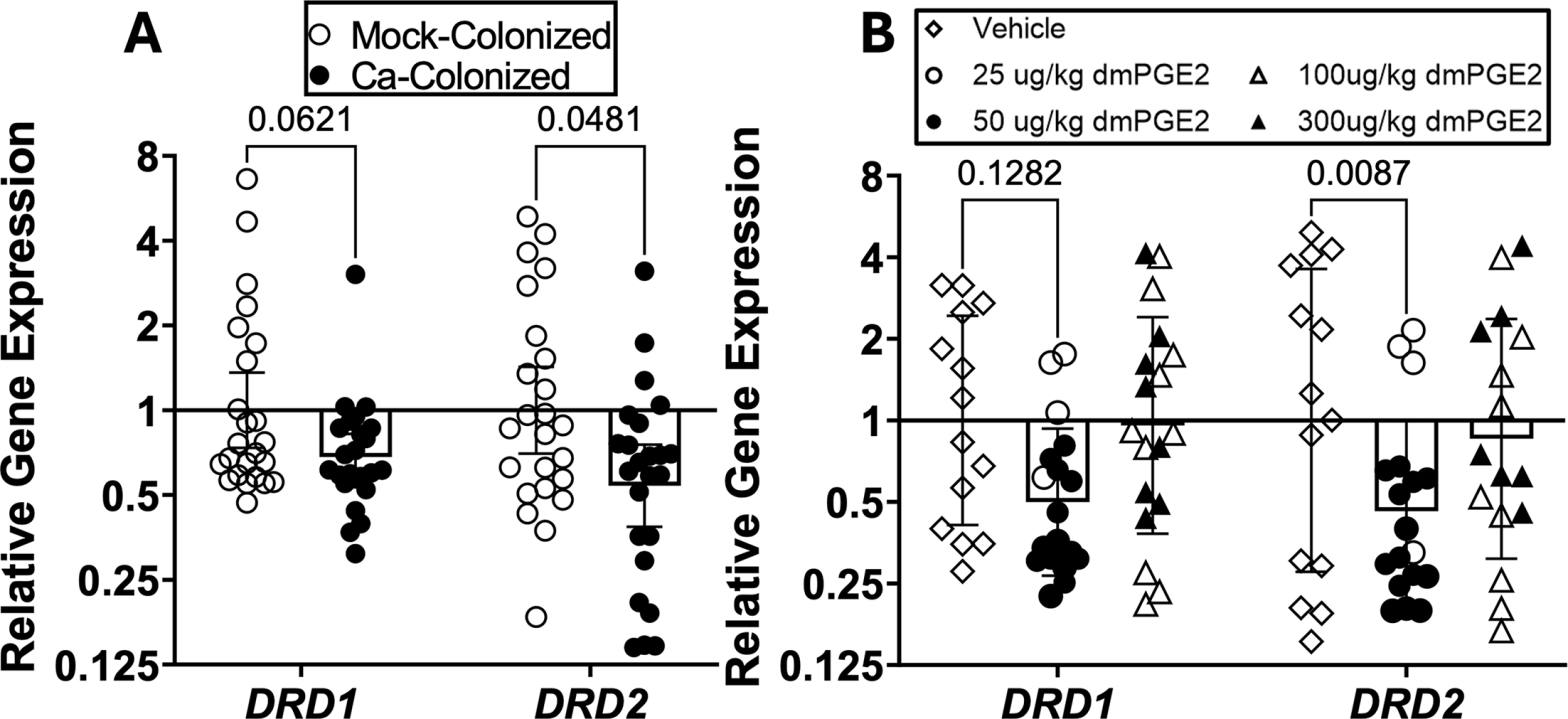
Both *C. albicans* colonization and low-dose dmPGE_2_ injection result in lower *Drd2* receptor expression in the dorsal striatum. (**A**) Single-housed female C57BL/6 mice were orally inoculated with *C. albicans* strain CKY101 or PBS and subjected to the two-bottle choice experiment as in Fig. 1. On day 2, mice were euthanized, and brains were collected. *Drd* receptor expression was measured in the dorsal striatum of mock-colonized or *C. albicans*-colonized mice by real-time RT-qPCR using the delta-delta CT (ddCT) method. (**B**) Single-housed female C57BL/6 mice were injected subcutaneously each day with dmPGE_2_ (low dose = 25 [open circles] or 50 µg/kg [closed circles]; high dose = 100 [open triangles] or 300 µg/kg [closed triangles]) or vehicle (open diamonds) and subjected to the two-bottle choice experiment as in Fig. 1. On day 4, mice were euthanized, and brains were collected. *Drd* receptor expression was measured in the DS by RT-qPCR using the ddCT method. Outliers were removed with ROUT to determine mathematical outliers (*Q* = 0.1% to remove definitive outliers). (**A, B**) Geometric means and geometric SDs are shown, and a two-way analysis of variance was performed for statistical significance.

We also analyzed the expression of dopamine receptors in the DS of these mice. The dopamine system in the DS is commonly involved with learning and habit formation (36). Expression of two main classes of dopamine receptors, *Drd1* and *Drd2*, was measured (37). A trend toward a reduction in *Drd1* expression and a statistically significant reduction in *Drd2* expression in *C. albicans*-colonized mice compared to mock-colonized mice was observed (Fig. 5A). Additionally, mice injected with low-dose dmPGE_2_ (25 or 50 µg/kg injections) showed reduced expression of *Drd2* (Fig. 5B). Interestingly, *Drds* showed no differences in expression in the PFC or NAc (Fig. S4). Furthermore, other addiction-related genes showed no significant differences in expression in the DS (Fig. S5). These results show that both *C. albicans* colonization and low-dose dmPGE_2_ injections reduce dopamine receptor expression in the DS.

**Fig 6:**
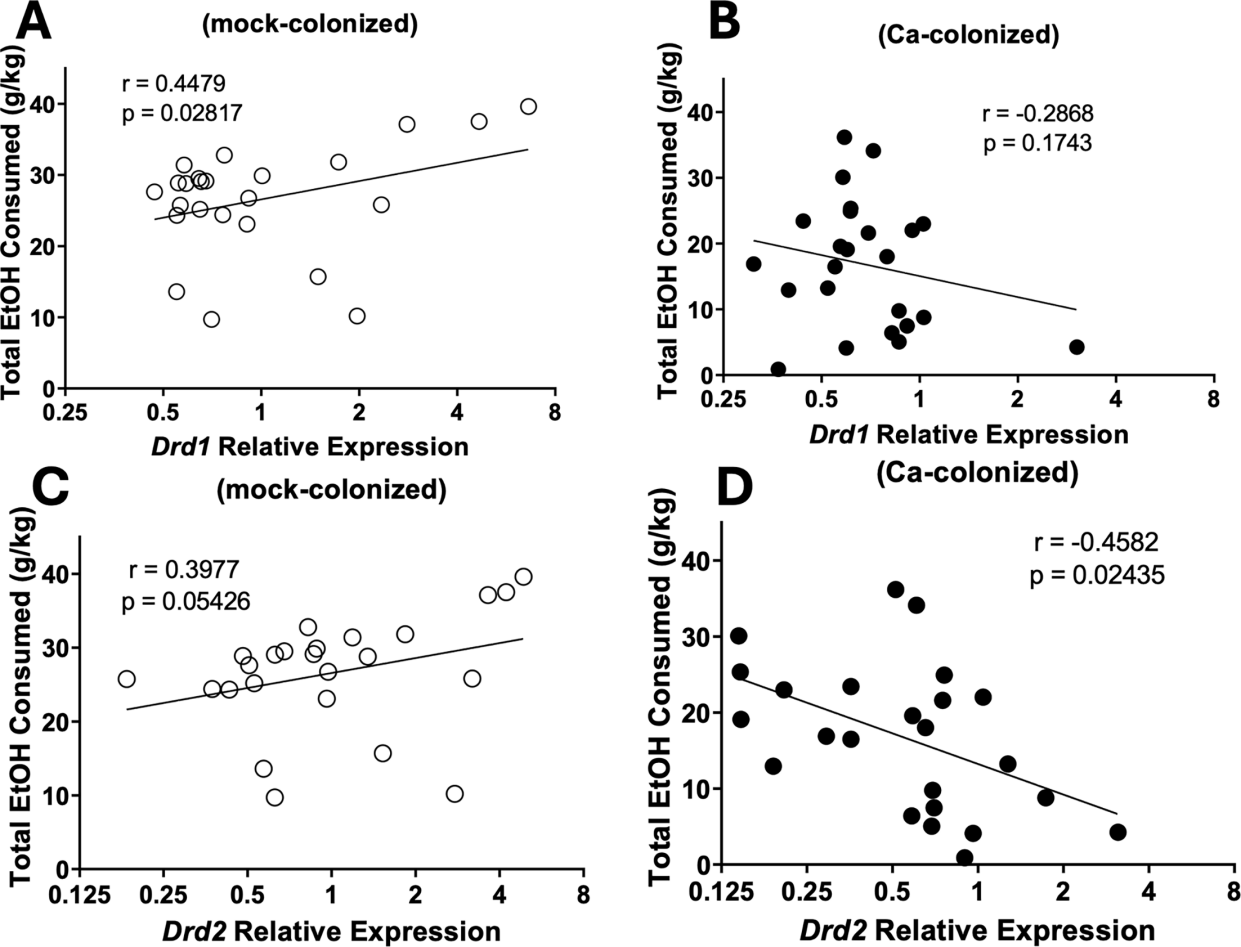
*Drd* expression correlates with ethanol consumption in mock- and *C. albicans*-colonized mice. Single-housed female C57BL/6 mice were orally inoculated with *C. albicans* strain CKY101 or PBS and subjected to the two-bottle choice experiment as in Fig. 1. On day 2, mice were euthanized, and brains were collected. *Drd* receptor expression was measured in the dorsal striatum of mock-colonized or *C. albicans*-colonized mice. Total ethanol consumption (grams per kilogram) is plotted as a function of relative *Drd* expression. Pearson correlation was used to test for significant correlations, *r* = correlation strength and *P* = statistical significance. (**A, C**) Mock-colonized mice. (**B, D**) *C. albicans-*colonized mice. (**A, B**) *Drd1* fold change of relative expression. (**C, D**) *Drd2* fold change of relative expression. Significant correlations between *Drd1* expression and ethanol consumption by mock-colonized mice and *Drd2* expression and ethanol consumption by *C. albicans*-colonized mice were observed.

Expression of *Drd1* or *Drd2* in mock- or *C. albicans*-colonized mice correlated with total ethanol consumed. In the dorsal striatum, DRD1 is involved with reward formation and DRD2 is involved with aversion formation (38). Plots of *Drd* expression and total ethanol consumed showed a significant positive correlation between *Drd1* expression and total ethanol consumption in mock-colonized mice (Fig. 6A) and a trend toward a positive correlation between *Drd2* and total ethanol consumed (Fig. 6C). In *C. albicans*-colonized mice, by contrast, a significant negative correlation between *Drd2* expression and total ethanol consumption was observed (Fig. 6D). No correlation between *Drd1* expression and total ethanol consumed was observed in these mice (Fig. 6B). Thus, *C. albicans-*colonized mice that consumed lower amounts of ethanol exhibited higher expression of the aversion-associated DRD2 receptor.

### *Candida albicans* colonization accelerates the onset of conditioned taste aversion in mice

Based on the low ethanol consumption and negative correlation between *Drd2* expression and ethanol consumption in *C. albicans*-colonized mice, we hypothesized that these mice would show enhanced ethanol-induced CTA. Ethanol-induced CTA is based on the principle that mice will develop an aversion to a novel tasting solution when it is paired with an ethanol injection (39, 40). Mice were tested for ethanol-induced CTA as described in Materials and Methods.

**Fig 7:**
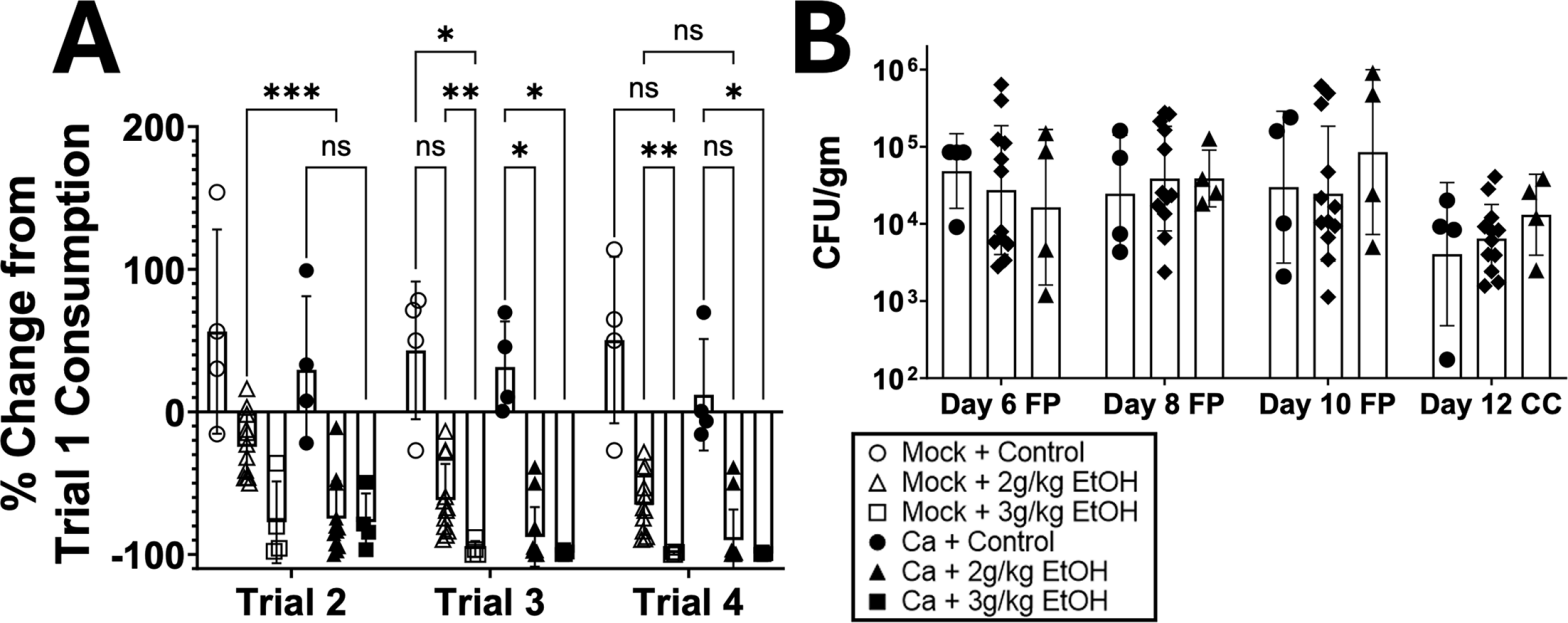
*C. albicans*-colonized mice develop conditioned taste aversion more rapidly than mock-colonized mice. Single-housed female C57BL/6 mice were trained to drink their daily liquid in the late morning. Mice orally inoculated with *C. albicans* strain CKY101 or PBS were given 1 hour of access to a novel tastant, 1.2% saline solution, and then injected intraperitoneally with ethanol (2 g/kg or 3 g/kg) or control (sterile saline). Tastant consumption on the first trial (before injection with ethanol) was used as the baseline measurement, and consumption on later trials was compared to trial 1. (**A**) percent change of tastant consumption relative to each individual mouse’s trial 1 consumption (pre-conditioned stimulus). Trial 1 corresponds to day 6 of the experiment, trial 2 corresponds to day 8 of the experiment, trial 3 corresponds to day 10 of the experiment, and trial 4 corresponds to day 12. (**B**) CFU per gram of *C. albicans* from fecal pellets collected on day 6, day 8, and day 10; cecum contents from day 12. (**A, B**) Mock-colonized, control (open circles); mock-colonized + 2 g/kg EtOH (open triangles); mock-colonized + 3 g/kg EtOH (open squares); *C. albicans*-colonized, control (closed circles); *C. albicans-*colonized + 2 g/kg EtOH (black triangles); *C. albicans-*colonized + 3 g/kg EtOH (closed squares). A two-way analysis of variance corrected for repeated measures was completed for statistical significance. ns, *P* < 0.0995; *P < 0.0499; **P < 0.0076; ***P = 0.0006.

The first exposure to the tastant showed the baseline consumption level, prior to injection (conditioning) with ethanol or control (sterile saline). Trials 2–4 showed consumption after conditioning, expressed as the change from initial consumption (Fig. 7A). In trial 2, control mice showed similar or higher-than-baseline consumption of the tastant. Both mock-colonized and *C. albicans*-colonized mice injected with 3 g/kg ethanol developed a near-complete aversion to the tastant (100% reduction), which continued through trials 3 and 4. In contrast, mice given a lower concentration of ethanol (2 g/kg) showed differences between groups. There was no significant aversion in the mock-colonized mice in trial 2 of this injection group, and *C. albicans*-colonized mice showed a significant reduction in saline consumption compared to control mice in trial 2. On trial 3, both mock-colonized and *C. albicans*-colonized mice injected with 2 g/kg ethanol showed significant aversion to the tastant. There were no differences in colonization or mouse weight throughout the experiment (Fig. 7; Fig. S6). *C. albicans-*colonized mice thus developed ethanol-induced CTA more rapidly.

### *C. albicans***-**colonized mice are more susceptible to the behavioral effects of ethanol, and motor coordination deficits can be restored by EP receptor antagonism

**Fig 8:**
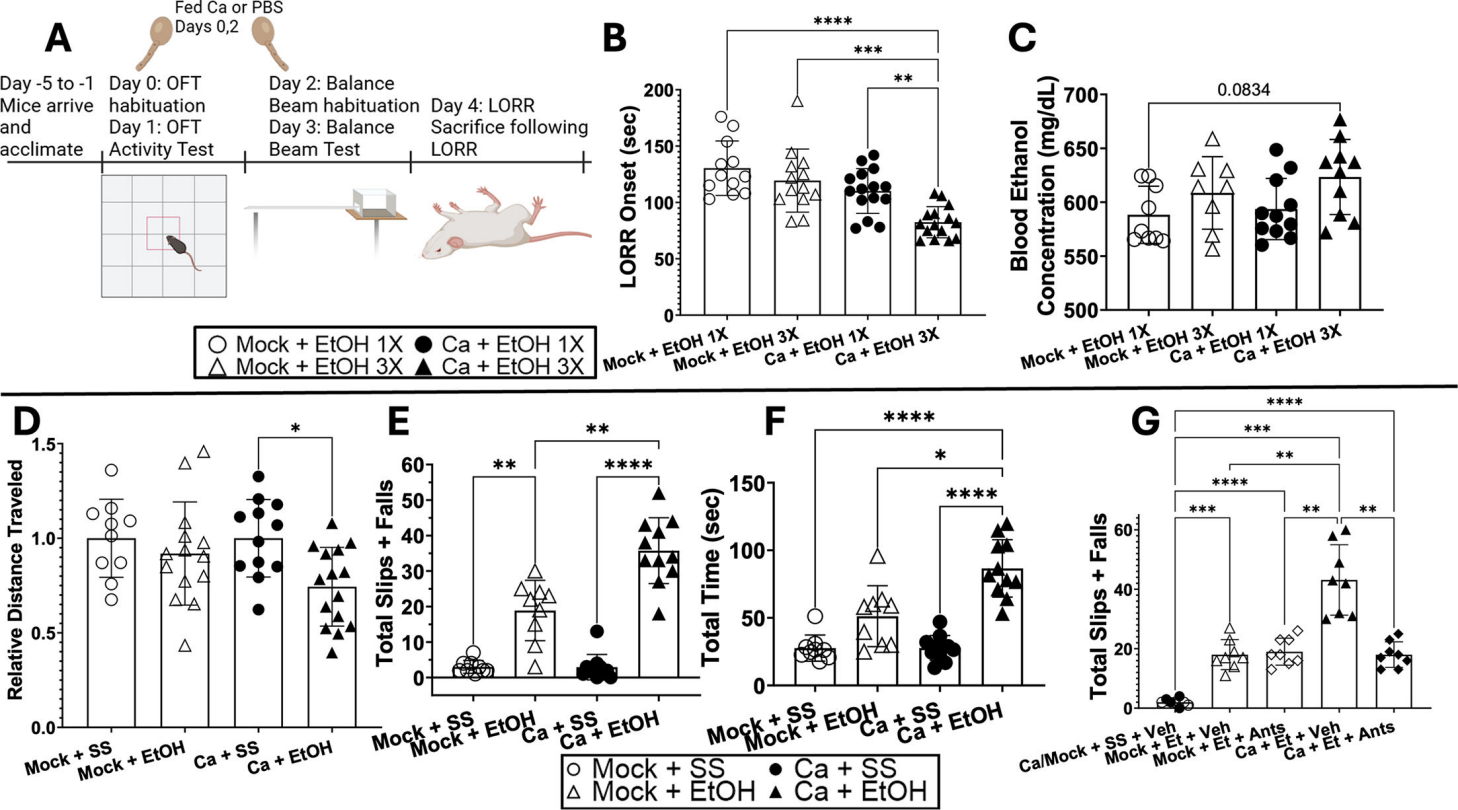
*C. albicans*-colonized mice are more sensitive to the effects of ethanol. Single-housed female C57BL/6 mice were orally inoculated with *C. albicans* strain CKY101 or PBS on days 0 and 2 and subjected to the behavioral tests illustrated in panel **A**. (**B, C**) Groups are defined as Mock + 1× EtOH: mock-colonized, received sterile saline ip injections on days 1 and 3 and 3.5 g/kg EtOH on day 4 (open circles); Mock + 3× EtOH: mock-colonized, received 1.5 g/kg ethanol ip injected on days 1 and 3 and 3.5 g/kg EtOH on day 4 (open triangles); Ca + 1× EtOH: *C. albicans*-colonized, received sterile saline ip injections on days 1 and 3 and 3.5 g/kg EtOH on day 4 (black circles); Ca + 3× EtOH: *C. albicans*-colonized, received 1.5 g/kg ethanol ip injected on days 1 and 3 and 3.5 g/kg EtOH on day 4 (black triangles). (**D–F**) Groups are defined as Mock + SS: mock-colonized, received sterile saline ip injections on days 1 and 3 (open circles); Mock + EtOH: mock-colonized, received 1.5 g/kg ethanol ip injected on days 1 and 3 (open triangles); Ca + SS: *C. albicans*-colonized, received sterile saline ip injections on day 1 and 3 (black circles); Ca + EtOH: *C. albicans*-colonized, received 1.5 g/kg ethanol ip injected on days 1 and 3 (black triangles). (**G**) Groups are defined as Ca/Mock + SS + Veh, fed *C. albicans* (black circles) or sterile PBS (open circles), received sterile saline and vehicle injections; Mock + Et + Veh, fed sterile PBS, received ethanol and vehicle injections; Mock + Et + Ants, fed sterile PBS, received ethanol and antagonist injections; Ca + Et + Veh, fed *C. albicans*, received ethanol and vehicle injections; Ca + Et + Ants, fed *C. albicans*, received ethanol and antagonist injections. All injections were IP, given before habituation or testing. Ethanol injections were 1.5 g/kg of mouse weight and antagonists were 2 g/kg of both EP1 and EP2 antagonists. (**B**) Time after 3.5 g/kg ethanol injection (seconds) for the mouse to lose its righting reflex (LORR onset). (**C**) Blood ethanol concentration (mg/dL) at the time of euthanization following LORR onset. (**D**) Distance traveled in the open field test in a 10-minute trial, shown relative to the average of the sterile saline-injected (SS) mice within the same group (mock-colonized or Ca-colonized). (**E**) Total number of foot slips and “falls” during three trials of the balance beam test. A foot slip was defined as the foot slipping beneath the balance beam, and a fall was defined as falling to the underside of the beam, or, on rare occasions, a fall off the beam. (**F**) Cumulative time required to cross the balance beam in three trials. (**G**) Total number of slips or “falls” from the EP-antagonist experiments. Mice were injected intraperitoneally with EP1 and EP2 antagonists or vehicle daily on days 0–4 approximately 1 hour before each behavioral test. (**B, C**) Ordinary one-way analyses of variance (ANOVAs) were completed for statistical significance. (**D–G**) Brown-Forsythe and Welch ANOVAs were completed for statistical significance. (**B–G**) Bars show means with the standard deviation or geometric mean with geometric standard deviation. Symbols represent individual mice. *P < 0.0220; **P < 0.0055; ***P < 0.0003; ****P < 0.0001.

The more rapid development of ethanol-induced CTA in *C. albicans*-colonized mice suggests these mice could be more susceptible to the behavioral effects of ethanol, as correlations between these phenotypes have been observed (41). Therefore, we tested whether *C. albicans*-colonized mice were more susceptible to ethanol sedation, ethanol-induced motor coordination defects, and depressant effects of ethanol (Fig. 8A).

The first experiment was the open field activity test (OFT), which measures how susceptible C57BL/6 mice are to the depressant effects of alcohol based on the decrease in distance traveled in the OFT following ethanol injection (1.5 g/kg) (42). Mice were injected with ethanol (dose) or control (sterile saline [SS]), and distance traveled in 10 minutes in the OFT was measured. Results are expressed relative to the SS-injected mice within each experimental trial (mock or *C. albicans*-colonized) (Fig. 8D). Results showed a significant reduction in distance traveled by *C. albicans*-colonized, ethanol-injected mice compared to *C. albicans*-colonized, SS-injected mice. There was not a significant reduction in distance traveled by the mock-colonized, ethanol-injected mice compared to the SS-injected mice. These results show that the *C. albicans*-colonized mice were more strongly affected by ethanol.

The loss of righting reflex (LORR) experiment tested the ability of the mice to right themselves after injection with a sedative amount of ethanol (3.5 g/kg) (43). The time for the mice to lose their righting reflex after the injection is termed LORR onset. Mock- or *C. albicans*-colonized mice that had received SS injections the previous 3 days or mock mice that received ethanol the previous 3 days had no differences in LORR onset (Fig. 8B). *C. albicans*-colonized mice that received ethanol injections on the previous 3 days showed a significantly shorter LORR onset compared to all groups. Blood ethanol concentration from serum collected at the time of loss of righting reflex showed a trend toward higher levels in these mice (Fig. 8C). These results indicate a synergistic effect of ethanol treatment (including prior treatment) and *C. albicans* colonization that increased the sedative effects of ethanol.

Motor coordination defects were tested using performance on the balance beam, which can measure coordination based on the number of slips and falls on the beam (44). Mice were injected with SS or ethanol, and their performance was assessed. The total number of slips or falls throughout three trials (Fig. 8E) and the total amount of time required to complete three trials were measured (Fig. 8F). Mock-colonized or *C. albicans*-colonized mice injected with SS did not slip/fall often and completed the trials quickly. Mock-colonized mice injected with ethanol showed a significant increase in slips and falls, consistent with ethanol-induced motor coordination deficits. *C. albicans*-colonized mice injected with ethanol showed significantly more slips and falls than any of the other groups and were significantly slower. Furthermore, there were no differences in colonization or mouse weight in these experiments (Fig. S7). Thus, *C. albicans*-colonized mice showed more ethanol-induced impairment in motor coordination.

To test the hypothesis that PGE_2_ signaling contributed to the differences in mouse susceptibility to the effects of ethanol, mice were injected with a combination of EP1 and EP2 antagonists (2 mg/kg each) before each behavioral test. Total slips and falls on the balance beam test following these injections were measured. *C. albicans*-colonized mice injected with ethanol and antagonists showed a significant decrease in slips and falls compared to the ethanol and vehicle group (Fig. 8G). There was no difference between the *C. albicans*-colonized group with ethanol and antagonists compared to either of the mock + ethanol-injected groups. We did not see a similar reversal of phenotypes by antagonists in either the OFT or LORR (Fig. S8). These results showed that blocking PGE_2_ signaling in *C. albicans*-colonized mice reduced their susceptibility to ethanol-induced impairment of motor control.

Taken together, the results showed that *C. albicans*-colonized mice were more susceptible to the depressant and sedative effects of ethanol, as well as to ethanol-induced impairment in motor control. PGE_2_ effects on EP1 and EP2 receptors make a major contribution to the mechanism of ethanol-induced motor impairment.

## DISCUSSION

We demonstrate that *C. albicans* colonization in mice reduces ethanol preference and consumption. Our results with EP1/EP2 antagonists show that PGE_2_ signaling plays a significant role in modifying mouse ethanol preference. Furthermore, injection of uncolonized mice with dmPGE_2_ reduced ethanol consumption. There was a somewhat dose-dependent response, as low concentrations of dmPGE_2_ (25 µg/kg–50 µg/kg) reduced consumption moderately and higher doses of dmPGE_2_ (100 µg/kg–300 µg/kg) reduced ethanol preference and consumption more strongly.

Antibiotic-treated mice with *C. albicans* gut colonization have been previously shown to exhibit elevated serum PGE-m levels by stimulating host macrophage production of PGE_2_ (25). It is currently unclear what fungal factors stimulate this increase *in vivo*. However, *C. albicans* stimulates host PGE_2_ production in immune cells via immunogenic cell wall components and can also synthesize its own PGE_2_ (45–47). Either of these mechanisms or both could contribute to the rise in serum PGE-m. Mice treated with antibiotics but not inoculated with fungi showed a modest increase in serum PGE-m levels, and this level was sufficient to promote inflammatory cell responses. *C. albicans*-colonized mice in our study, which did not use antibiotics, also showed modestly increased serum concentrations of PGE-m, and PGE-m concentration correlated with ethanol preference. These results indicate that modest increases in serum PGE_2_ are associated with changes in host immune responses and behavior. PGE_2_ in circulation may be directly involved in these changes or may serve as a marker indicating higher levels of PGE_2_ in multiple organs. In fact, we saw evidence of increased PGE_2_ by correlations with *Ep* expression in all regions of the brain examined.

PGE_2_ has previously been shown to alter the permeability of the blood-brain barrier (BBB) by inducing alterations in endothelial permeability or by stimulating the retraction of pericytes from the surface of the BBB (48–50). PGE_2_ has also been shown to induce vasodilation (48). Vasodilatory effects and BBB alterations could lead to higher amounts of PGE_2_ in the brain and reduce ethanol consumption. These results support the model that, as PGE_2_ in the circulation elevates above normal fluctuating ranges, BBB permeability increases, and more PGE_2_ can enter the brain, resulting in correlations between its concentration in serum and *Ep1/Ep2* receptor expression in *C. albicans*-colonized mice. It is also possible that there are additional changes other than PGE_2_ levels that regulate BBB permeability in these mice, such as changes in levels of immune factors or endocannabinoids. Alterations to levels of endocannabinoids have been previously shown to alter BBB permeability (51), and our lab has previously shown that *C. albicans*-colonized mice have alterations to the endocannabinoid system (ECS) (26). Immunological or metabolic changes could also contribute to the observed phenotypes of *Ep* and *Drd* receptor expression changes in *C. albicans*-colonized mice but not in mock-colonized mice.

Synergy between the effects of *C. albicans* colonization and the effects of ethanol was also observed. Mice that received both ethanol and *C. albicans* were the only group with significantly reduced locomotion in the OFT. *C. albicans*-colonized mice that received multiple injections with ethanol showed significantly shorter time to LORR onset than *C. albicans*-colonized mice that received one injection. Ethanol also affects the BBB through changes in integrity and permeability (52, 53). Inflammatory molecules such as lipopolysaccharide (LPS) can also synergistically increase BBB permeability (54). The effects of ethanol on the BBB may thus synergize with the effects of PGE_2_, resulting in significant behavioral effects in colonized mice. Saccharin preference may not be reduced by *C. albicans* colonization because of the lack of an additional insult to the BBB.

PGE_2_ has previously been shown to affect the dopamine system. Dopamine is critical for habit, reward, and aversion formation (55). PGE_2_ treatment altered reward learning to morphine in mice (56, 57) and has been shown to alter DRD signaling and behavior following cocaine ingestion (58). In this latter study, PGE_2_ is shown to increase DRD signaling intensity. Therefore, an extracellular milieu with higher PGE_2_ could lead to DRD-expressing neurons that require lower expression of dopamine receptors to yield similar activity. High-dose dmPGE_2_-injected mice that have similar expression of dopamine receptors compared to vehicle-injected mice could have a greater activity of DRD2-expressing neurons leading to stronger aversion phenotypes. We observed that *C. albicans*-colonized mice showed altered expression of dopamine receptors in the dorsal striatum. Expression of one of the receptors, *Drd2*, was significantly decreased in *C. albicans*-colonized mice and low-dose dmPGE_2_-injected mice. Interestingly, expression in the PFC and NAc did not indicate similar changes to the dopamine system, and other genes implicated in addiction such as neuropeptide Y receptors, opioid receptors (Mu and Delta), proopiomelanocortin (*Pomc*), and *Fos* were unchanged. Significant expression differences of the Mu opioid receptor between mock- and *C. albicans*-colonized mice appeared to be due to differences in ethanol consumption. These results indicate an altered regulation of the dopamine system in the DS observed in mice with low-level increases in systemic PGE_2_ concentrations.

Furthermore, when *Drd* expression in the DS was plotted against total ethanol consumption, significant positive or negative correlations were observed. DRD1 and DRD2 receptors in the dorsal striatum have well-defined, consistent roles. DRD1 signaling in the DS is mostly involved with reinforcement or reward learning, and DRD2 is most commonly involved with aversion formation (38). A positive correlation between *Drd1* expression and total ethanol consumption was observed in mock-colonized mice, but not in *C. albicans*-colonized mice, suggesting dysregulation of the relationship of DRD1/reinforcement in these mice. Additionally, a negative correlation between *Drd2* expression and total ethanol consumption in *C. albicans*-colonized mice suggested that aversion to ethanol could develop. Therefore, there could be a lack of reinforcement learning, an active aversion, or a combination of both that leads to lower ethanol preference in *C. albicans*-colonized mice. Results from the CTA suggest an aversion-dominated phenotype because colonized mice showed a more rapid acquisition of conditioned taste aversion. These findings support the conclusion that aversion to ethanol develops more readily in *C. albicans*-colonized mice.

The dorsal striatum was the main focus of these experiments due to its role in habit formation (34). However, it is possible that there are also changes to other dopaminergic and dopaminoceptive regions of the brain. For example, with changes observed in the expression of *Drd* receptors in the striatum, it is possible that there are significant changes to the dopaminergic regions that project to the striatum, such as the ventral tegmental area and the substantia nigra (59). These regions also project to the PFC and amygdala, which could alter behaviors such as fear- or stress-dependent learning (59, 60) in *C. albicans*-colonized mice. Changes to the dopaminergic regions of the brain would suggest broader circuit-wide changes impacting a wide range of behaviors. Focus on these regions and other impacted behaviors in *C. albicans*-colonized mice will be the subject of future research.

When conducting other behavioral tests, we found that *C. albicans*-colonized mice injected with ethanol showed increased LORR onset, decreased locomotion in the OFT, and further impaired performance on the balance beam. The performance on the balance beam could be restored to mock-colonized performance by EP1/EP2 antagonists. These results suggest that PGE_2_ impact on EP1 and EP2 receptors could contribute to motor control following ethanol intoxication. The depressant and sedative effects of ethanol detected in the OFT and LORR tests could be driven by other systems impacted by *C. albicans* colonization, such as the ECS (26). Both therapeutic manipulation and gene knockouts of the ECS modulated LORR and OFT phenotypes (61–63). Effects of *C. albicans* colonization on this pathway (26) could play a role in these phenotypes.

All of the experiments described in this communication were done with ethanol-naïve mice with short-term colonization by *C. albicans*. The effects of chronic *C. albicans* colonization on mice chronically exposed to ethanol are unknown. It is currently unclear how the results of this study relate to AUD, a chronic condition, and this unknown is a limitation of the study. There could be an altered effect of *C. albicans* colonization on the host in the setting of AUD and/or chronic stress. Low DRD2 receptor abundance has been shown in individuals with AUD and predisposes mice to stress-induced increases in ethanol consumption (64, 65). If DRD2 receptor abundance is reduced in humans with *C. albicans* colonization, as it is in colonized mice, these individuals may consume more ethanol under stress, possibly exacerbating ethanol consumption in AUD. However, our study focused only on initial alcohol consumption, and these effects of *C. albicans* colonization in individuals with AUD and/or chronic stress are currently unstudied. Additionally, the effects of native commensals of mice such as *Kazachstania pintolopesii* or common foodborne yeasts such as *Saccharomyces cerevisiae* on mouse ethanol consumption remain unclear. These are important areas for future research.

In summary, we have shown that GI colonization by *C. albicans* elevates serum PGE_2_, and these changes correlate with changes in *Ep* and *Drd* receptor expression in the dorsal striatum. *C. albicans* colonization decreased ethanol consumption and preference in C57BL/6 mice. These decreases in ethanol consumption correlated with changes in dopamine receptor expression, and *C. albicans*-colonized mice showed altered behavioral effects of ethanol. In conclusion, we show for the first time that changes in the fungal microbiome can impact host ethanol consumption and behavioral responses to ethanol.

## MATERIALS AND METHODS

### Animals

Five-week-old female or male C57BL/6 mice (Jackson Laboratory) were acclimated to the facility and single housing for 4–5 days with scruffing to acclimate to handling. Mice were orally inoculated with *C. albicans* at different times depending on the experiment. Mice were inoculated by pipetting 20 µL containing 5 × 10^7^ cells of *C. albicans* strain CKY101 (66) in PBS (cultured in yeast extract, peptone, dextrose broth at 30°C; nearly all cells were in yeast form), or PBS only for mock-colonized mice, directly into the mouth of the mouse as previously described (26). Mock-colonized mice were given 20 µL of PBS directly into their mouths. Mice were monitored throughout the experiment for colonization as previously described (26). No fungal colonies were observed in mock-colonized mice.

Mouse weight was measured throughout the experiment. Rarely, if a mouse lost 20% of its starting body weight, the mouse was euthanized. On the last day of each experiment, mice were euthanized in the afternoon. Mice were anesthetized using the isoflurane drop method and decapitated with a rodent guillotine. Blood for serum and various organs was harvested for analysis.

### Chemicals and reagents

The EP1 antagonist SC-51089 (Cayman Chemical) and the EP2 antagonist TG6-10-1 (Cayman Chemical) were resuspended in a 2:3:5 mixture of dimethyl sulfoxide (DMSO):sterile water:PEG400. Antagonists were resuspended and mixed at a concentration that would yield 2 mg/kg of each antagonist in 50 µL, and 50 µL of the mixture was injected intraperitoneally.

Fifteen percent ethanol was prepared for consumption by diluting 95% ethanol (Fisher) with tap water. This solution was filter-sterilized and added to sipper tubes for the two-bottle choice experiment.

16,16-Dimethyl prostaglandin E_2_ (Cayman Chemical) was dried under a gentle stream of nitrogen to evaporate the solvent. It was then resuspended in DMSO and injected into mice subcutaneously at a concentration of 25, 50, 100, or 300 µg/kg.

### Two-bottle choice ethanol or saccharin consumption

During acclimation to the facility and single housing, mice also began acclimating to the sipper tubes used in the two-bottle choice experiment. Briefly, sterile acidified water was poured into 50 mL conical tubes (USA Scientific), and a sterile stopper (#6 Rubber, Ancare) with a sipper spout (Ancare) was placed into the 50 mL conical tube and wrapped with parafilm. Two of these sipper bottles with water were placed into standard cages for acclimation.

Following acclimation, mice were weighed and assigned to groups with equal weights. Mice were inoculated with *C. albicans* or mock-inoculated on day −1. On day 0, 15% (vol/vol) ethanol or 0.0125% (wt/vol) saccharin replaced one water bottle. Mouse weight and tube weight were then measured daily throughout the experiment. Sipper bottles were rotated daily to minimize side preference. After 2–4 days of ethanol consumption, mice were euthanized as previously described.

For ethanol preference, results show 26 mice per group from three replicate experimental trials. For saccharin preference, 14–15 mice per group from two experimental trials are shown.

### PGE-m ELISA

Trunk blood from mice was obtained after decapitation and collected in BD blood collection tubes (365967, Fisher). Tubes were then centrifuged in an Eppendorf table-top microcentrifuge at 10,000× *g* for 10 minutes, and serum was stored at −80°C. Serum was assayed with a Prostaglandin E Metabolite ELISA Kit (514531, Cayman) after removal of proteins by acetone precipitation. PGE-m ELISA was performed using the standard protocol, with derivatization, as detailed in the product manual. Serum was diluted 15–25 times for the assay, and OD was measured on a Biotek Epoch2 plate reader. A four-parameter logistic curve was determined, and raw PGE-m values were determined using the 4PL calculator by AAT Bioquest (67).

### Brain dissection, RT-qPCR, and primers

Mouse brains were dissected, flash-frozen on aluminum foil chilled with dry ice, and kept at −80°C. A brain matrix (ZIVIC Instruments, 5325) was allowed to chill on ice for 30 minutes. Two millimeter sections of the brain, from directly anterior of the cerebellum to the frontal pole, were then cut using clean straight razor blades and the brain matrix. Sections were then observed to find landmarks indicating the location of the nucleus accumbens, prefrontal cortex, and dorsal striatum, as previously described (68, 69). These structures were dissected, and RNA was extracted as previously described (26).

cDNA was then synthesized using random hexamer or oligo dT primers, and RT-qPCR was performed as previously described (26). Primer sequences are listed in Table S1.

### Ethanol-induced conditioned taste aversion

Following arrival, mice were acclimated to single housing and sipper tubes by placing one sipper tube of water in their cages. Mice were weighed every other day to ensure they were consuming enough water to maintain their weight. The following protocol was adapted from a previous protocol that showed significant conditioned taste aversion in C57BL/6 mice (70). During the first 4 days, mice were acclimated to sipper tubes and single housing. Then, mice were subjected to 5 days of water restriction, with only 2 hours of access to water daily (termed days 1–5 of the experiment). On day 5 of water restriction, mice were inoculated with *C. albicans* or PBS for mock colonization and were re-inoculated every 2 days (days 5, 7, 9, 11). On day 6, mice were given access to 1.2% saline for 1 hour in their home cages and injected with sterile saline, 2 g/kg of ethanol, or 3 g/kg of ethanol (20% vol/vol) immediately after the end of the 1 hour period. Five hours later, mice were given access to water for 30 minutes to rehydrate. This process was repeated for days 8 and 10; on day 12, mice were given access to 1.2% saline for 1 hour in their home cages and then euthanized. Fecal pellets were collected for CFU determination on days 6, 8, and 10, and cecum contents were collected on day 12. Data were analyzed as a percentage of saline consumption compared to the initial amount consumed on day 6.

### Open field activity test

On the OFT habituation day, mice were injected an average of 15 minutes before their respective trials with 100 µL of sterile saline intraperitoneally using an insulin syringe. Following 15 minutes, mice were gently placed in the center of a 37 cm × 37 cm plastic open field box. Mice were allowed to roam freely for 10 minutes and removed from the apparatus. The apparatus was then sterilized using 70% ethanol. This protocol was repeated for every mouse.

On OFT day, mice were injected with 1.5 g/kg of ethanol or sterile saline intraperitoneally an average of 15 minutes before their trials. The volume of injection varied from approximately 90 µL–110 µL depending on the weight of the mouse. The ethanol concentration was adjusted so that a mouse of average weight for the group would be injected with 100 µL ethanol solution. The 10 minute test run and cleaning of the apparatus were repeated as above for every mouse. Mouse behavior was recorded using a web camera. Scoring videos was performed using EthovisionXT software to automatically determine distance traveled. Total distance traveled was plotted relative to the mean distance traveled by the sterile saline group.

### Balance beam

A balance beam apparatus was constructed using a square 0.25 inch diameter, 3-foot-long metal rod held approximately 1.5 ft above the surface. A plastic box to hold bedding was affixed to one end to provide incentive for the mouse to cross the entirety of the balance beam. A reading lamp shining on the start zone of the beam was used as an aversive stimulus. Start and end points for the balance beam trials spanning 65 cm of the beam were marked.

On habituation day, mice were injected with 100 µL of sterile saline intraperitoneally approximately 15 minutes before the start of their first of three trials. Mice were then placed in the start zone to begin trial 1. After completing the trial, mice were given 30 seconds of rest in their home cage before the start of trial 2. Trials 2 and 3 were performed using the same procedure. After a mouse completed three trials, the balance beam was cleaned using 70% ethanol, and the next mouse was tested. In the event that a mouse would freeze, or very rarely fall, it would be gently prodded or placed back on the bar until it completed the trial. All mice within a cohort underwent this procedure.

On test day, mice were injected with 100 µL of sterile saline or injected with 1.5 g/kg of ethanol as described in the open field activity test section. These injections were performed approximately 15 minutes before the start of the first trial. A web camera was used to record mouse behavior during these trials. The three trials were repeated as described above. All mice completed the entirety of the three trials.

Videos were blinded for scoring. Start and stop times were determined when the mouse’s nose crossed designated marks on the bar. A slip was determined when a mouse’s foot slipped below the bar or a significant slip from a standing position on the bar to the pelvis touching the bar occurred. A fall was scored when the mouse slipped to the underside of the bar. Very rarely a mouse would fall from the bar into safety padding below the bar. Both of these events were scored equally as falls. Time or slips and falls were summed for all three trials for each mouse.

### Loss of righting reflex

Mice were co-housed with another mouse within the same group for the LORR. Their nestlet was removed for the test. Mice were injected with 3.5 g/kg of ethanol intraperitoneally, and the time of injection was recorded. Within roughly 1–2 minutes, most mice lost righting reflex, which was defined as an inability to right themselves when lying on their back in a V-shaped plastic trough. The time when a mouse lost righting reflex was designated the LORR onset. Six minutes after injection, mice were given 1 minute of exposure to isoflurane via the drop method and euthanized as above. Occasionally, a mouse would have to be removed from the data due to shearing of an artery during injection—significantly altering LORR time.

### Blood ethanol concentration assay

Following LORR, trunk blood was collected, and serum was extracted as previously described in “PGE-m ELISA” above. For the blood ethanol concentration determination, an EnzyChrom Ethanol Assay Kit (ECET-100, BioAssay Systems) was used. Standard protocols were followed for the determination using serum that was diluted 15×. OD was measured on a Biotek Epoch2 plate reader.

## SUPPLEMENTAL MATERIAL

Supplemental material mbio.02239-25-s0001.docx Figures S1 to S8; Table S1.

ASM does not own the copyrights to Supplemental Material that may be linked to, or accessed through, an article. The authors have granted ASM a non-exclusive, world-wide license to publish the Supplemental Material files. Please contact the corresponding author directly for reuse.
