# Supplementary material for: Candida albicans colonization modulates murine ethanol consumption and behavioral responses through elevation of serum prostaglandin E2 and impact on the striatal dopamine system
Source: mBio. 2025 Oct 16;16(11):e02239-25. doi: 10.1128/mbio.02239-25 (PMC12607880; doi:10.1128/mbio.02239-25)
Supplement: Supplemental material — Figures S1 to S8; Table S1. [file mbio.02239-25-s0001.docx]

**Day et al Supplemental Information**

**
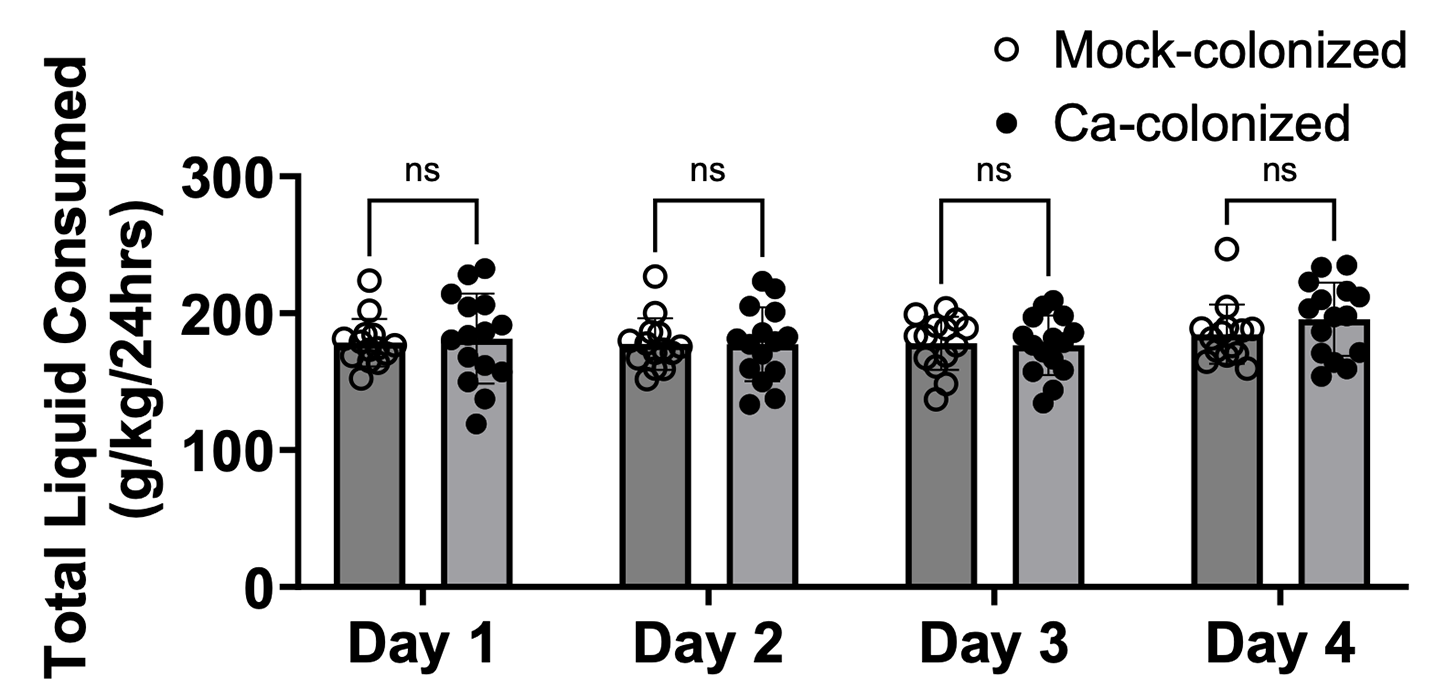
**

***Figure S1: No difference in total liquid consumed by C. albicans-colonized mice and mock-colonized mice when ethanol was not present.*** Single housed female C57BL/6 mice were orally inoculated with *C. albicans* strain CKY101 or PBS and subjected to the saccharin preference test from **Figure 1**. Total liquid consumed was tracked by day as total grams of liquid consumed/kilogram of body weight. A Two-way ANOVA was completed for statistics and no significant differences were observed. Mock-colonized mice are shown in open circles and *C. albicans*-colonized mice are shown in closed circles. Bar shows the mean.

**
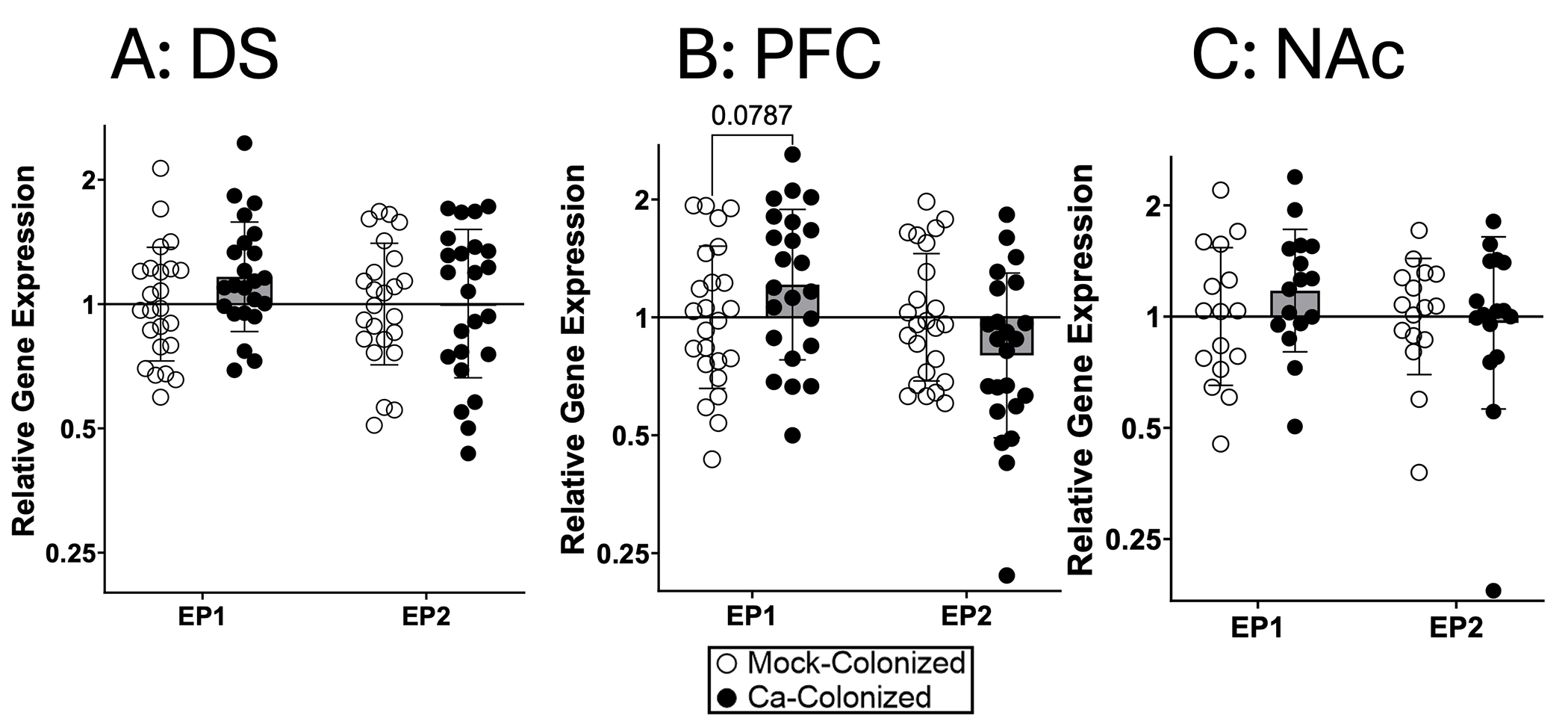
**

***Figure S2: Ep receptor expression were not different in C. albicans-colonized mice vs mock-colonized mice in various brain regions.*** Single housed female C57BL/6 mice were orally inoculated with *C. albicans* strain CKY101 or PBS and subjected to the 2-bottle choice experiment as in Figure 1. On day 2, mice were euthanized, and brains were collected. *Ep* receptor expression was measured in various brain regions of mock-colonized or *C. albicans*-colonized mice by RT-qPCR using the ddCT method. Geometric mean and geometric standard deviation are shown. A Two-way ANOVA was performed for statistics and p-values are shown. **(A)** Shows expression in dorsal striatum (DS) **(B)** Shows expression in the prefrontal cortex (PFC) **(C)** Shows expression in the nucleus accumbens (NAc)

***
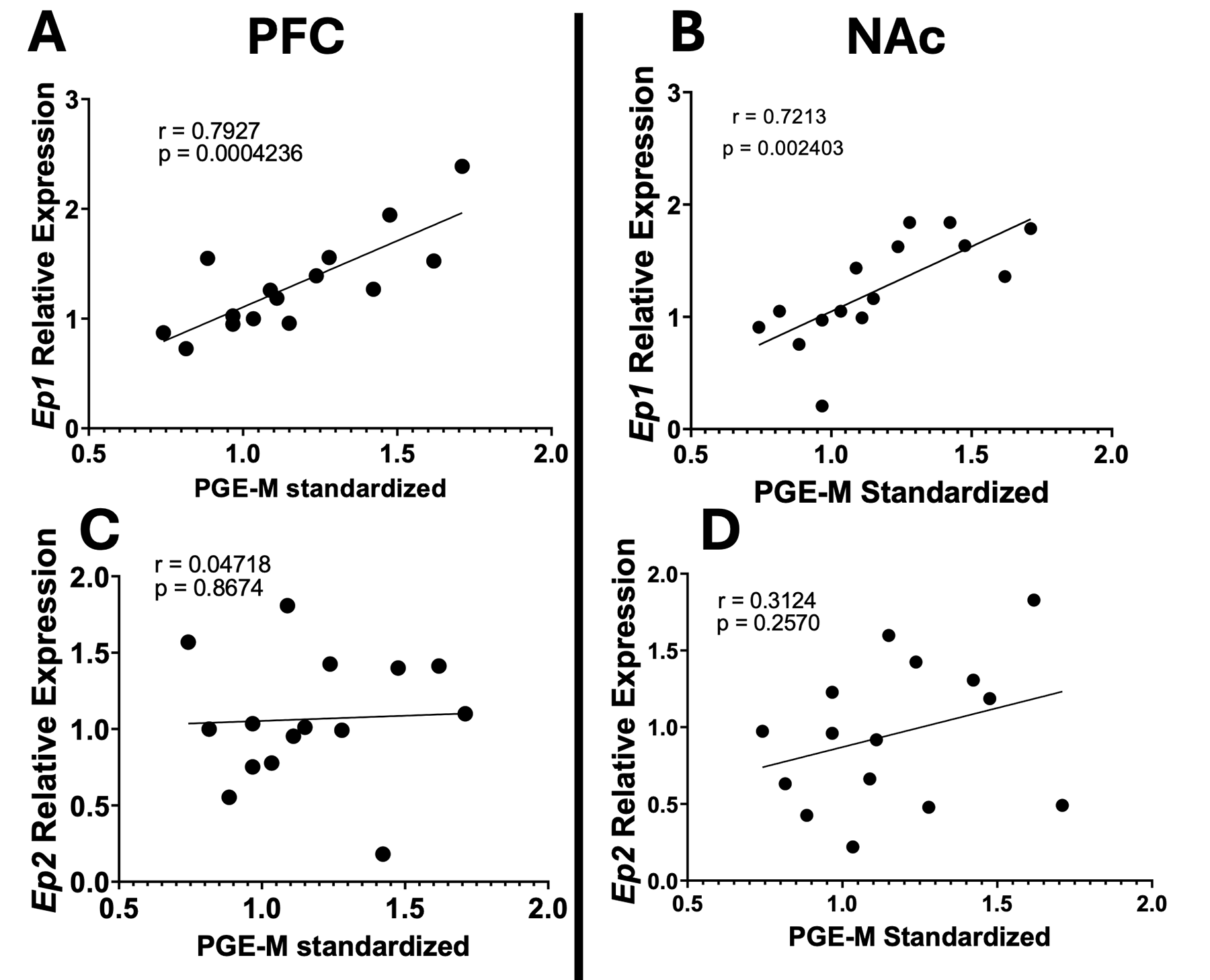
***

***Figure S3: Serum PGE-metabolite correlates with EP1 Receptor expression in the PFC and NAc of C. albicans-colonized mice.*** Single housed female C57BL/6 mice were orally inoculated with *C. albicans* strain CKY101 or PBS and subjected to the 2-bottle choice experiment as in Figure 1. On day 2, mice were euthanized, and brain and serum were collected. Expression of *Ep* genes relative to average expression in mock-colonized mice from the same experimental trial, plotted as a function of concentration of PGE-metabolite standardized to the average in mock-colonized mice in the same experimental trial. Each symbol represents an individual mouse. **(A,C)** show gene expression of *Ep1* and *Ep2*, respectively, in the prefrontal cortex (PFC). **(B,D)** show gene expression of *Ep1* and *Ep2*, respectively, in the nucleus accumbens (NAc). Pearson correlations were used to test for significant correlations, r=correlation strength and p=statistical significance. Significant correlations were observed between *Ep1* expression and concentration of PGE-M in the PFC and NAc of *C. albicans-*colonized mice.

***
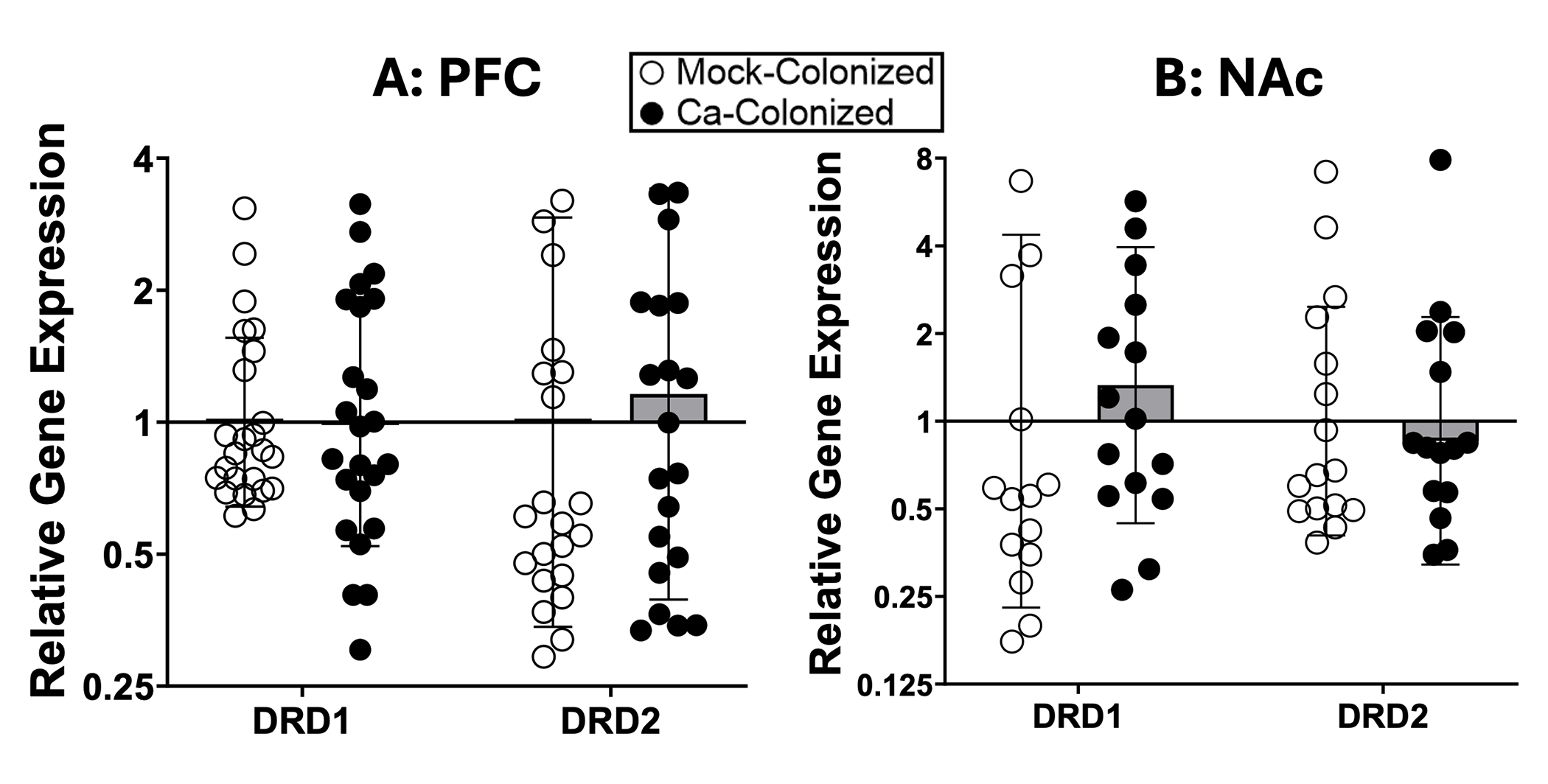
***

***Figure S4: Drd gene expression was not different between C.* *albicans-colonized and mock-colonized mice in the PFC and NAc.*** Single housed female C57BL/6 mice were orally inoculated with *C. albicans* strain CKY101 or PBS and subjected to the 2-bottle choice experiment as in Figure 1. On day 2, mice were euthanized, and brains were collected. *Drd* receptor expression was measured in the prefrontal cortex (PFC) **(A)** or nucleus accumbens (NAc) **(B)** of mock-colonized or *C. albicans*-colonized mice by RT-qPCR using the ddCT method. Geometric mean and geometric standard deviation are shown. Multiple t-tests performed for statistics—no significant differences were observed.

***
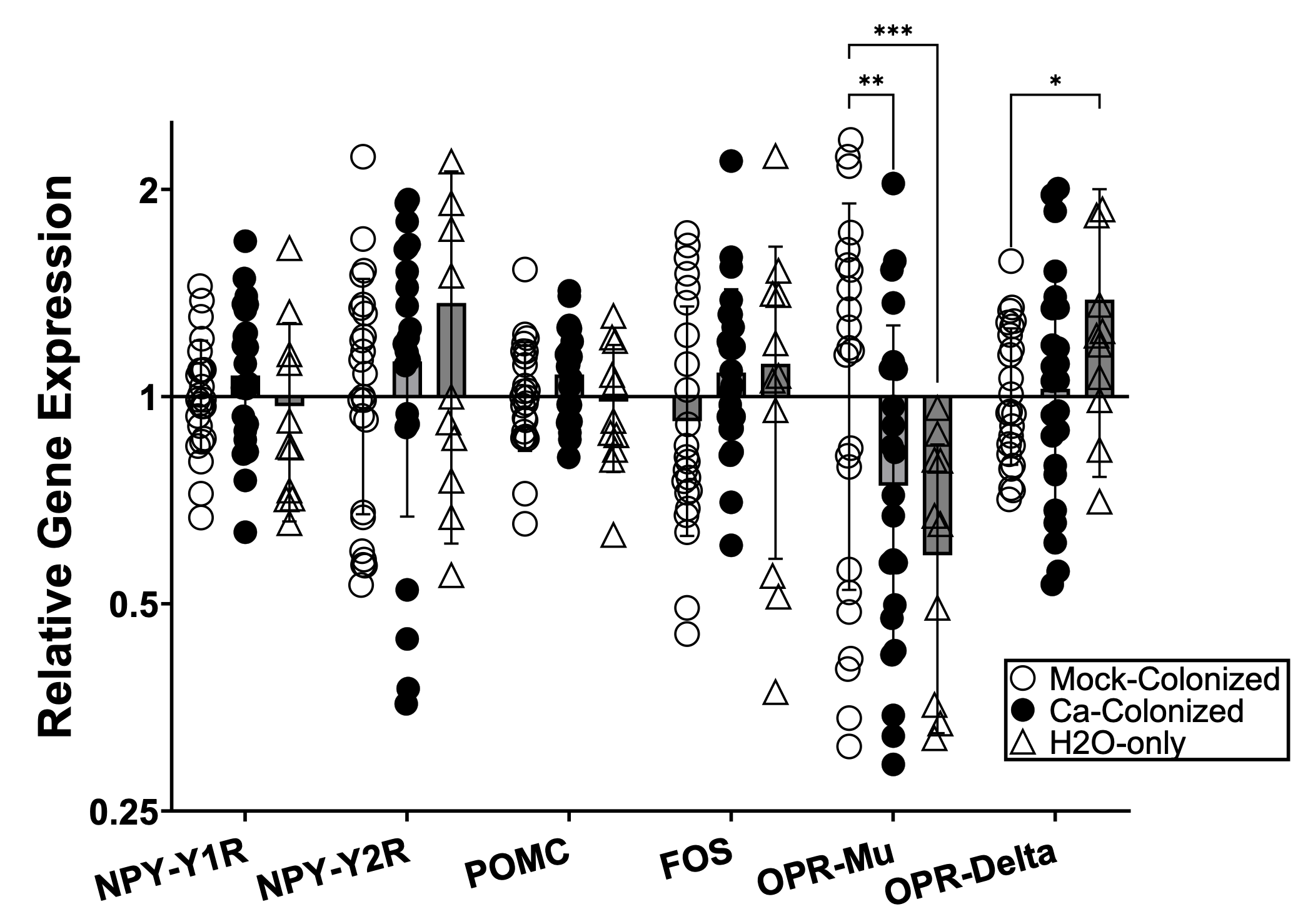
***

***Figure S5: Expression of other addiction-implicated genes in the Dorsal Striatum of C. albicans-colonized mice***. Single housed female C57BL/6 mice were orally inoculated with *C. albicans* strain CKY101 or PBS and subjected to the 2-bottle choice experiment as in Figure 1. On day 2, mice were euthanized, and brains were collected. Groups of mice that consumed ethanol (Ca-colonized or Mock-colonized) or mice that were mock-colonized and only given access to water (H2O-only) are shown. The expression of genes implicated in addiction was measured in the dorsal striatum of the different groups by RT-qPCR using the ddCT method. Geometric mean and geometric standard deviation are shown. A two-way ANOVA with Dunnett’s correction was performed for statistics. * p=0.0315; ** p=0.0065; *** p=0.0001.

***
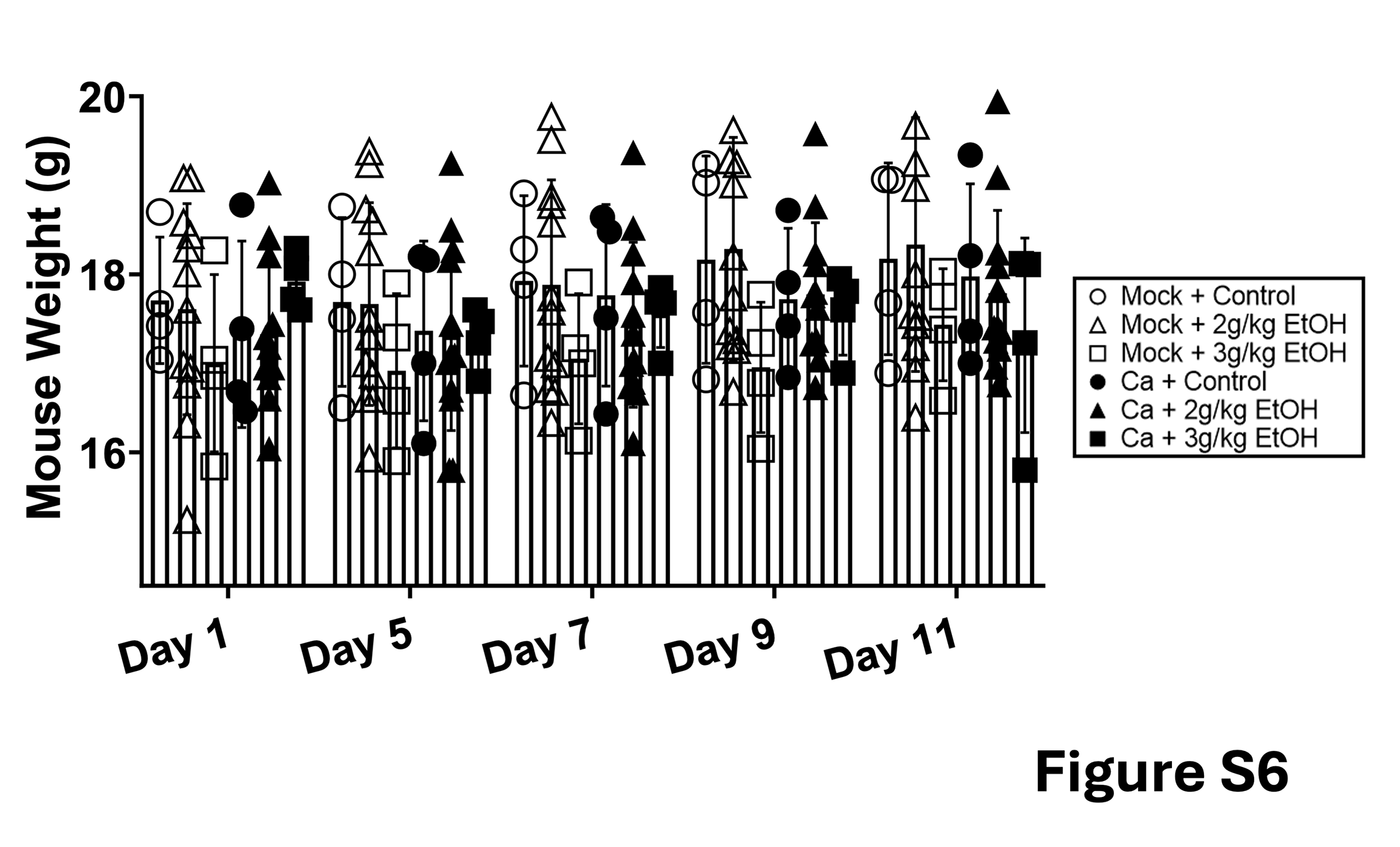
***

***Figure S6: No differences in mouse weight were observed in the ethanol-induced conditioned taste aversion experiment***. Single housed female C57BL/6 mice were trained to drink their daily liquid in the late morning. Mice orally inoculated with *C. albicans* strain CKY101 or PBS were given one hour of access to a novel tastant, 1.2% saline solution, and then injected intraperitoneally with ethanol (2 g/kg or 3 g/kg) or control (sterile saline). Mouse weight is shown in grams. Mock-colonized, control (open circles); mock-colonized + 2g/kg EtOH (open triangles); mock-colonized + 3 g/kg EtOH (open squares); *C. albicans*-colonized, control (closed circles); *C. albicans-*colonized +2 g/kg EtOH (black triangles); *C. albicans-*colonized + 3 g/kg EtOH (closed squares). Bars show means, error bars show standard deviation, and each symbol represents one mouse. A two-way ANOVA corrected for repeated measures was performed for statistical significance—there were no significant comparisons.

***
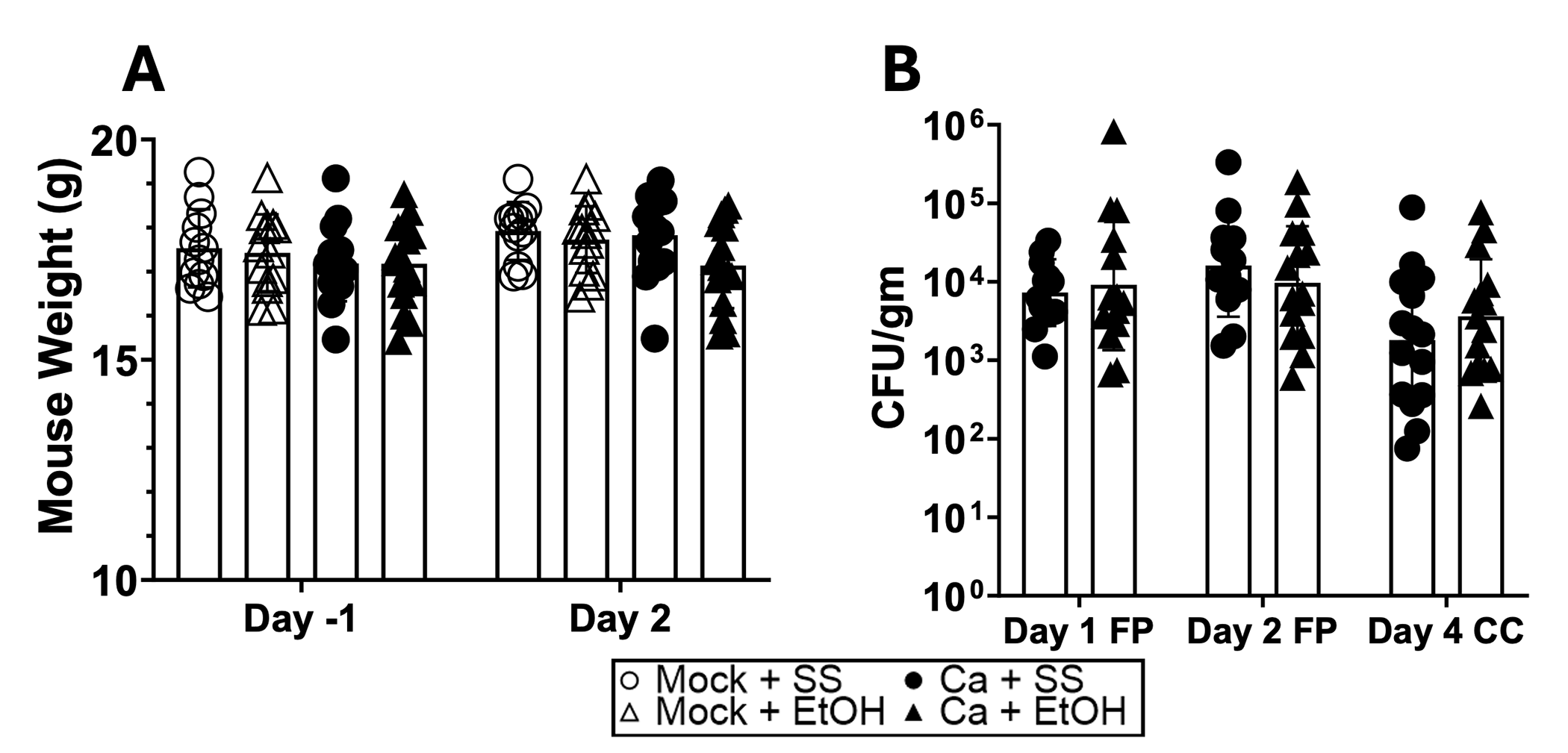
***

***Figure S7: No differences in mouse weight or colonization were observed in the LORR, OFT, and balance beam experiments.*** Single housed female C57BL/6 mice were orally inoculated with *C. albicans* strain CKY101 or PBS on days 0 and 2, and subjected to the behavioral tests illustrated in **Fig. 8A. (A)** Mouse weights on days -1 and 2 are shown. **(B)** CFU/gm fecal pellets (FP) or cecum contents (CC) collected on days indicated. Bars show means with the standard deviation (A) or geometric mean with geometric standard deviation (B). Symbols represent individual mice. Two-way ANOVAs corrected for multiple repeated measures were performed for statistics and there were no significant differences.

***
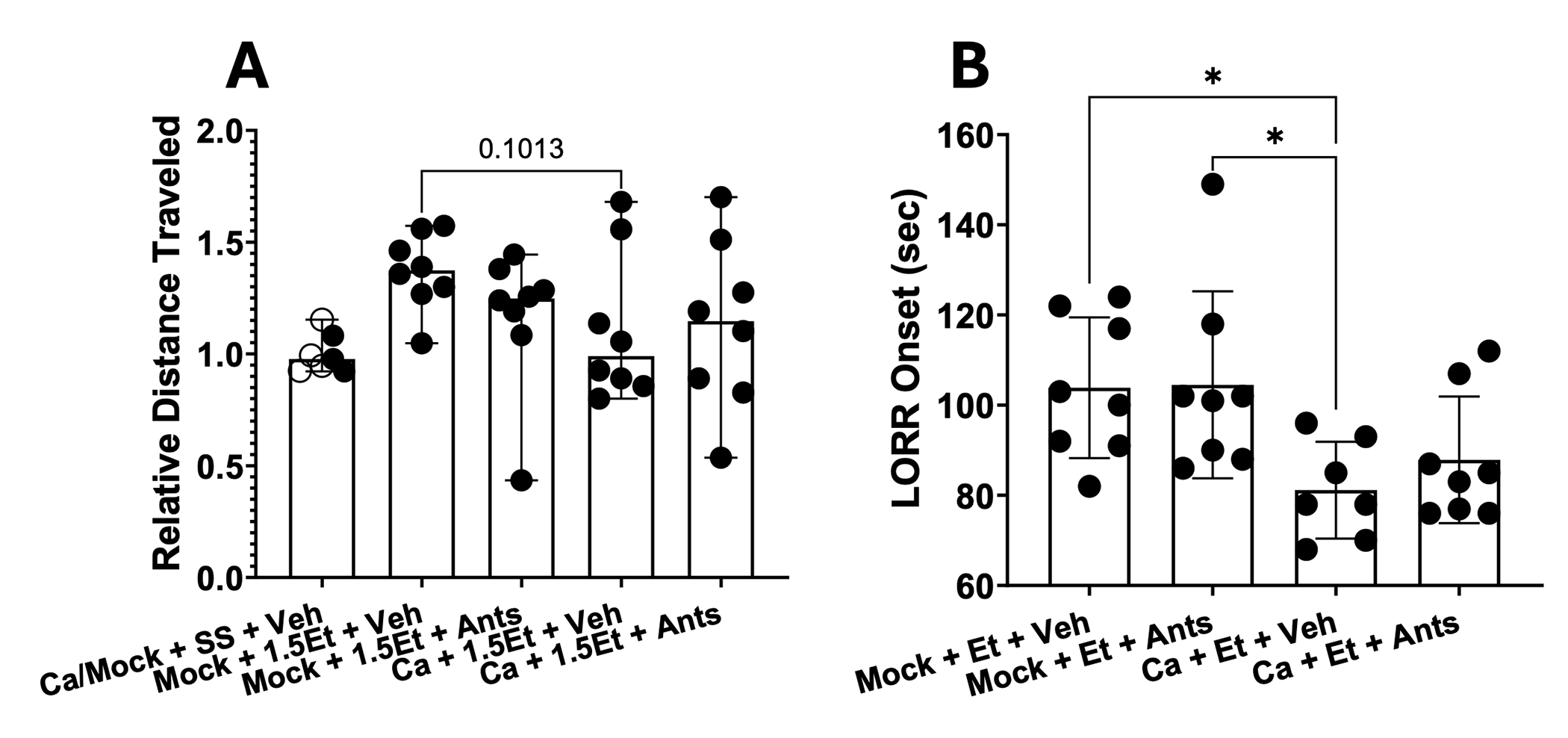
***

***Figure S8: Antagonism of Ep receptors did not change the behavior of mice in the OFT or LORR tests.*** Single housed female C57BL/6 mice were orally inoculated with *C. albicans* strain CKY101 or PBS on days 0 and 2, and subjected to the behavioral tests illustrated in **Figure 8A**, except that mice were injected intraperitoneally with EP1 and EP2 antagonists or vehicle daily on days 0-4 approximately one hour before each behavioral test. **(A)** Groups are defined as: Ca/Mock + SS + Veh: mock-colonized or *C. albicans-*colonized, received sterile saline ip injections on days 1 and vehicle ip injections daily; Mock + EtOH + Veh: mock-colonized, received 1.5g/kg ethanol ip injected on days 1 and vehicle ip injections daily; Mock + EtOH + Ants: mock-colonized, received 1.5g/kg ethanol ip injected on days 1 and antagonist ip injections daily; Ca + EtOH + Veh: *C. albicans*-colonized, received 1.5g/kg ethanol ip injected on days 1 and vehicle ip injections daily; Ca + EtOH + Ants: *C. albicans*-colonized, received 1.5g/kg ethanol ip injected on days 1 and antagonist ip injections daily **(B)** Groups are defined as: Mock + Et + Veh: mock-colonized, received 1.5g/kg ethanol ip injected on days 1 and 3 and 3.5g/kg EtOH on day 4 and vehicle daily; Mock + Et + Ants: mock-colonized, received 1.5g/kg ethanol ip injected on days 1 and 3 and 3.5g/kg EtOH on day 4 and antagonists daily; Ca + Et + Veh: *C. albicans*-colonized, received 1.5g/kg ethanol ip injected on days 1 and 3 and 3.5g/kg EtOH on day 4 and vehicle daily; Ca + Et + Ants: *C. albicans*-colonized, received 1.5g/kg ethanol ip injected on days 1 and 3 and 3.5g/kg EtOH on day 4 and antagonists daily. **(A)** distance traveled in the open field test in a ten-minute trial relative to the average for sterile saline-injected (SS) mice within the same group (mock-colonized or *C. albicans*-colonized). A Brown-Forsythe and Welch ANOVA test was run for statistical significance. **(B)** time after injection (seconds) for the mouse to lose its righting reflex (LORR onset). An ordinary One-way ANOVA was completed for statistical significance. ***** p<0.0465.

***Table S1: Primers used In this study***

| **Primer Name for RT-qPCR** | **Sequence** | **Source** |
| --- | --- | --- |
| GAPDH-FW | TGTAGACCATGTAGTTGAGGTCA | S1. |
| GAPDH-RV | AGGTCGGTGTGAACGGATTTG |  |
| EP1-FW | TGCTTGCCATCGACCTAGC | S2. |
| EP1-RV | CACCCAGGAAATGACACGC |  |
| EP2-FW | CAGCTCGGTGATGTTCTCGG | S2. |
| EP2-RV | GAGCACCAATTCCGTTACCAG |  |
| NPY-Y1R-FW | GACTCTCACAGGCTGTCTT | S3. |
| NPY-Y1R-RV | TTGGTCTCACTGGACCTGT |  |
| NPY-Y2R-FW | TTTTCGGAGGCTACCAATGT | S3. |
| NPY-Y2R-RV | AATACAATGGGAGGTCTGCA |  |
| POMC-FW | GAGGCCTTTCCCCTAGAGTT | S4. |
| POMC-RV | CACCGTAACGCTTGTCCTT |  |
| FOS-FW | GGGACAGCCTTTCCTACTAC | S5. |
| FOS-RV | GGGATAAAGTTGGCACTAGAG |  |
| OPR-MU-FW | GAGCCACAGCCTGTGCCCT | S6. |
| OPR-MU-RV | CGTGCTAGTGGCTAAGGCATC |  |
| OPR-DELTA-FW | GCTCGTCATGTTTGGCATC | S6. |
| OPR-DELTA-RV | AAGTACTTGGCGCTCTGGAA |  |
| DRD1-FW | GAACCCAGAAGACAGGTGGA | S7. |
| DRD1-RV | GCTTAGCCCTCACGTTCTTG |  |
| DRD2-FW | TATGCCCTGGGTCGTCTATC | S7. |
| DRD2-RV | AGGACAGGACCCAGACAATG |  |

**Supplemental Bibliography:**

S1. Markey L, Hooper A, Melon LC, Baglot S, Hill MN, Maguire J, et al. Colonization with the commensal fungus Candida albicans perturbs the gut-brain axis through dysregulation of endocannabinoid signaling. Psychoneuroendocrinology. 2020 Nov;121:104808.

S2. Chen L, Ji X, Wang M, Liao X, Liang C, Tang J, et al. Involvement of TLR4 signaling regulated-COX2/PGE2 axis in liver fibrosis induced by Schistosoma japonicum infection. Parasites Vectors. 2021 Dec;14(1):279.

S3. Shi Y ‐C., Ip CK, Reed F, Sarruf DA, Wulff BS, Herzog H. Y5 receptor signalling counteracts the anorectic effects of PYY 3‐36 in diet‐induced obese mice. J Neuroendocrinology. 2017 Oct;29(10):e12483.

S4. Luque RM, Gahete MD, Hochgeschwender U, Kineman RD. Evidence that endogenous SST inhibits ACTH and ghrelin expression by independent pathways. American Journal of Physiology-Endocrinology and Metabolism. 2006 Aug;291(2):E395–403.

S5. Almeida A, Paul Thiery J, Magdelénat H, Radvanyi F. Gene expression analysis by real-time reverse transcription polymerase chain reaction: influence of tissue handling. Analytical Biochemistry. 2004 May;328(2):101–8.

S6. Reiss D, Ceredig RA, Secher T, Boué J, Barreau F, Dietrich G, et al. Mu and delta opioid receptor knockout mice show increased colonic sensitivity. European Journal of Pain. 2017 Apr;21(4):623–34.

S7. Braunstein KE, Eschbach J, Ròna-Vörös K, Soylu R, Mikrouli E, Larmet Y, et al. A point mutation in the dynein heavy chain gene leads to striatal atrophy and compromises neurite outgrowth of striatal neurons. Human Molecular Genetics. 2010 Nov 15;19(22):4385–98
